# Atomic-level engineering and imaging of polypeptoid crystal lattices

**DOI:** 10.1073/pnas.1909992116

**Published:** 2019-10-21

**Authors:** Sunting Xuan, Xi Jiang, Ryan K. Spencer, Nan K. Li, David Prendergast, Nitash P. Balsara, Ronald N. Zuckermann

**Affiliations:** ^a^Molecular Foundry, Lawrence Berkeley National Laboratory, Berkeley, CA 94720;; ^b^Materials Sciences Division, Lawrence Berkeley National Laboratory, Berkeley, CA 94720;; ^c^Department of Chemistry, University of California, Irvine, CA 92697;; ^d^Department of Chemical Engineering & Materials Science, University of California, Irvine, CA 92697;; ^e^College of Chemistry, University of California, Berkeley, CA 94720

**Keywords:** peptoid polymers, cryo-TEM, supramolecular assembly, polymer amphiphiles, nanosheets

## Abstract

Rational design of supramolecular nanomaterials fundamentally depends upon an atomic-level understanding of their structure and how it responds to chemical modifications. Here we studied a series of crystalline diblock copolypeptoids by a combination of sequence-controlled synthesis, cryogenic transmission electron microscopy, and molecular dynamics simulation. This family of amphiphilic polypeptoids formed free-floating 2-dimensional monolayer nanosheets, in which individual polymer chains and their relative orientations could be directly observed. Furthermore, bromine atom side-chain substituents in nanosheets were directly visualized by cryogenic transmission electron microscopy, revealing atomic details in position space inaccessible by conventional scattering techniques. While the polypeptoid backbone conformation was conserved across the set of molecules, the nanosheets exhibited different lattice packing geometries dependent on the aromatic side chain *para* substitutions. Peptoids are inherently achiral, yet we showed that sequences containing an asymmetric aromatic substitution pattern pack with alternating rows adopting opposite backbone chiralities. These atomic-level insights into peptoid nanosheet crystal structure provide guidance for the future design of bioinspired nanomaterials with more precisely controlled structures and properties.

## Significance

A fundamental challenge in materials science is to understand the atomic-level structures of nanoarchitectures assembled from synthetic polymers. Here, we report a family of sequence-defined polypeptoids that form free-floating crystalline 2-dimensional nanosheets, in which not only individual polymer chains and their relative orientations, but also atoms in nanosheets were directly observed by cryogenic transmission electron microscopy. These atomic details are inaccessible by conventional scattering techniques. Using the feedback between sequence-controlled synthesis and atomic imaging, we observed how the nanosheet structure responds to chemical modifications at the atomic-length scale. These atomic-level insights open the door to the design of bioinspired nanomaterials with more precisely controlled structures and properties.

Synthetic polymer-based 2-dimensional (2D) nanomaterials have large surface area-to-volume ratios and can be produced with highly tunable chemical diversity, which gives them promise as platforms for filtration, catalysis, and sensing as well as components in semiconductors, circuits, and photovoltaics (1, 2). However, despite ongoing advances in design, synthesis, and characterization, it is still a significant challenge to generate high-aspect-ratio 2D structures with atomic precision across micrometer (or greater) length scales.

Poly(*N*-substituted glycines) (a.k.a. polypeptoids) are a family of nonnatural sequence-defined polymers that have received growing attention as a platform to create biomimetic nanomaterials (3). Compared to polypeptides, polypeptoids (in which the side chain is appended to the amide nitrogen rather than the alpha carbon) lack hydrogen-bond donors and chiral centers along their backbones. This leads to excellent thermal processability, good solubility in common solvents, and enhanced resistance toward enzymatic and hydrolytic degradation (4, 5). The reduced structural complexity means that the peptoid properties and structure are dominated by the side-chain identity and monomer sequence, which simplifies their design and engineering. In addition, the efficient, iterative submonomer solid-phase synthesis method, using primary amines as synthons, allows the incorporation of enormous side-chain diversity and precise control over monomer sequence and chain length (4, 6). Polypeptoids can form ordered 2D materials with crystal-like packing of monomers into a hierarchical assembly (3, 7, 8). All these attributes, combined with their similarity to polypeptides in biocompatibility and potent biological activities, make polypeptoids an attractive material for self-assembly of 2D materials with biomedical and materials science relevance. Polypeptoid nanosheets have already shown promise as antibody-mimetic scaffolds for molecular recognition (9, 10), self-repairing membrane mimetics (7, 11), and templates for biomineralization (12).

**Fig. 1.**
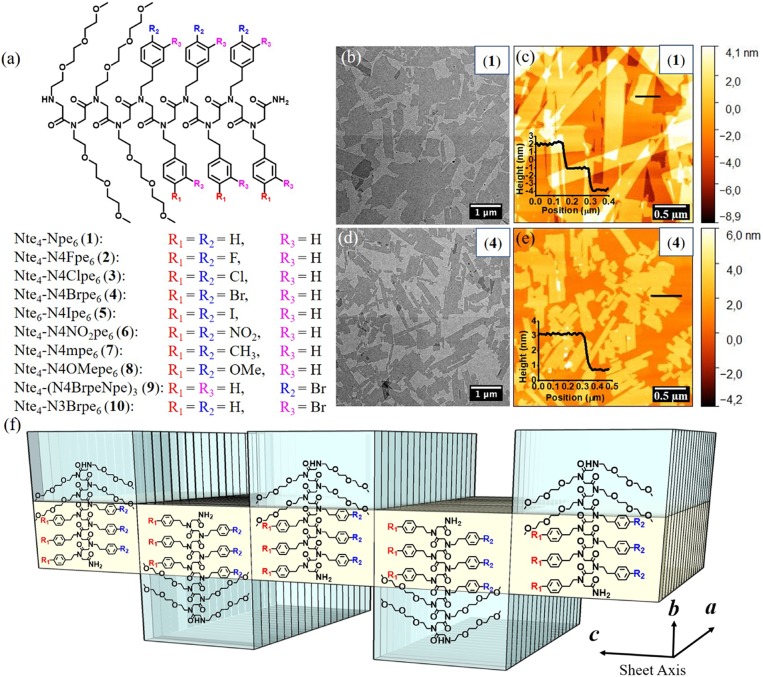
Assembly of amphiphilic diblock copolypeptoids into nanosheets via evaporation of a THF/water solution. (*A*) Chemical structures of diblock copolypeptoids **1** through **10**. The Nte block of compound **5** was increased to 6 monomers to increase the water solubility of this peptoid. (*B* and *D*) Representative TEM images of nanosheets. (*C* and *E*) Representative AFM images of nanosheets. The inset graphs are thickness profiles of nanosheets. (*F*) Proposed nanosheet structures of amphiphilic diblock copolypeptoids: Polypeptoid chains are packed antiparallel along the *c* direction and parallel along the *a* direction. The hydrophobic block (yellow color) is crystalline and the hydrophilic block (blue color) is amorphous.

Polypeptoid 2D nanosheet crystals can be made from sequences that contain a small set of amphiphilic monomers, arranged either in an alternating (3, 13) or diblock sequence pattern (7). Amphiphilic diblock copolypeptoids can form nanosheets in high yields by simply dissolving them in organic solvent/water mixture followed by subsequent evaporation of the organic solvent to induce the crystallization of the hydrophobic block (7, 8, 11). Amphiphilic diblock copolypeptoids of poly(*N*-decylglycine)-*b*-poly(*N*-2-(2-(2-methoxyethoxy)ethoxy)ethylglycine) (Ac-Ndc-Nte) (8) and poly(*N*-4-chlorophenylethylglycine)-*b*-poly(*N*-carboxyethylglycine) (N4Clpe-Nce) (7, 11) have been shown to form highly crystalline 2D nanosheets using this evaporation method. Despite the efforts on investigating the structures of the diblock copolypeptoid nanosheets by a variety of techniques (e.g., X-ray scattering, atomic force microscopy [AFM], transmission electron microscopy [TEM], and molecular dynamics [MD] simulation), a comprehensive understanding of the nanosheet structures on the atomic length scale is lacking. In the reported studies of diblock copolypeptoid nanosheets, it was proposed that polypeptoid chains are packed in rectangular lattices antiparallel along the *c* direction and parallel in the *a* direction (Fig. 1*F*) (7, 8, 11). X-ray scattering revealed that the nanosheet lattice has a universal *a* spacing between adjacent backbones (∼4.5 Å) along the *a* direction, while the *c* spacing between adjacent backbones in the *c* direction varied depending on the chemistry of the side chains. However, scattering techniques, by their very nature, provide information across many unit cells in the material, and the data are obtained in reciprocal space, not in position space. Therefore, X-ray scattering alone cannot reveal atomic details of heterogeneity within a polymer crystal lattice. So far there has been no method capable of revealing how the side-chain chemistry (e.g., size and electron distribution, etc.) can affect the atomic-level packing interactions in polypeptoid assemblies. In order to rationally design the nanosheets with more precise control over their properties and structures, it is crucial to fully understand their crystal structures in atomic detail and investigate the impact of side-chain chemistry and sequence on their atomic-level structures.

To elucidate the atomic-level structures of nanosheets, direct visualization of the individual chains within the nanosheet crystals is needed. Recent advances in low-dose cryogenic TEM (cryo-TEM) allow high-resolution imaging of radiation-sensitive biological macromolecules such as proteins with minimum electron beam damage (14–16); however, it remains a significant challenge to image synthetic soft materials, such as polymer crystals, at atomic resolution due to their inherent structural heterogeneity. In our recent study, high-resolution images of crystalline diblock copolypeptoid nanosheets were obtained with direct visualization of crystalline grains and grain boundaries on atomic length scales, using a combination of crystallographic and single-particle methods developed for cryo-TEM of biological macromolecules (8). Briefly, the electron micrographs were divided into small boxes comprising unit cells, which were then classified and averaged to reveal the shape and positioning of individual chains, and to map out the distribution of structural heterogeneity in the crystalline nanosheet. However, due to the substantial heterogeneity of those samples, the atomic model matching the major crystal motifs observed by cryo-TEM has not been established in our previous study (8). To obtain more detailed atomic-scale structural information, polypeptoid nanosheet crystals with high atomic-level homogeneity are desired. Additionally, we aimed to investigate the effect of side-chain chemistry on the structure of crystalline nanosheets by engineering the electron-dense heavy atoms (e.g., Br or I) to particular locations in the crystal lattice. The increased contrast in low-dose EM micrographs enables the direct imaging of heavy atoms after image processing.

In this contribution, we designed and synthesized a series of amphiphilic diblock copolypeptoids with the same hydrophilic poly(*N*-2-(2-(2-methoxyethoxy)ethoxy)ethylglycine) block (Nte) and a series of *N*-2-phenylethylglycine-based hydrophobic blocks bearing a systematic series of aromatic ring substituents, varying in size and electron withdrawing or donating character. We synthesized analogs with hydrogen, fluorine, chlorine, bromine, iodine, nitro, methyl, and methoxy substituents at the *para* position of the ring, and bromine substituent at the *meta* position of the ring, and examined the impact of these substituents on the crystal packing in the lattice. We previously demonstrated that *ortho* substituents are not tolerated in *N*-(2-phenylethyl)glycine–containing peptoid nanosheets (13). All these diblock copolypeptoids (except the one with the nitro substituent) formed 2D nanosheet crystals in water. The atomic-scale structures of the crystalline nanosheets and the effect of aromatic side-chain substituents on their crystal packing were studied by low-dose cryo-TEM and X-ray scattering. The combination of sequence control offered by submonomer peptoid synthesis, atomic-level imaging, and MD simulations provides a powerful platform to structurally engineer synthetic materials with the same precision as found in protein engineering. The atomic-level insights revealed in the polypeptoid crystals in this study will open more fundamental questions on structure–property relationships and provide the foundation upon which to direct the rational design of polypeptoid 2D materials with atomic precision.

## Results and Discussion

### Design and Synthesis of Amphiphilic Diblock Copolypeptoids.

Polypeptoids lack chiral centers and -NH···O = C- hydrogen bonding along the backbone, which results in self-assembly governed predominantly by interactions between side chains. Our goal is to elaborate the atomic structure of the polypeptoid nanosheet crystal lattice, without disrupting their packing motif, by making subtle variations to the side-chain structure and monomer sequence. We chose to examine diblocks based on the *N*-(2-phenylethyl)glycine (Npe) monomer with a 4:6 block composition of 4 Nte monomers and 6 Npe monomers as this sequence motif was established as one of the shortest known crystalline nanosheet-forming sequences (7). We then explored their packing rules by varying the *para* substituents of the aromatic side chains. Thus, a series of sequence-defined diblock copolypeptoid decamers containing a hydrophilic block of poly(*N*-2-(2-(2-methoxyethoxy)ethoxy)ethylglycine) (Nte) and a hydrophobic aromatic block bearing different *para*-substituted 2-phenylethyl side chains were synthesized by solid-phase submonomer synthesis (Fig. 1*A*). A series of *para* substituents, ranging in size and containing both electron-withdrawing groups (F, Cl, Br, I, and NO_2_) and electron-donating groups (CH_3_ and OMe) were chosen. The compound with bromine at the *meta* position of the phenyl side chains was also synthesized to investigate the effect of substitution position on the formation of nanosheets and the crystal lattice packing. All of the polypeptoids were purified by reverse-phase high-performance liquid chromatography to obtain ≥95% molecular purity. Our ability to directly image peptoid nanosheet lattices with atomic resolution by cryo-TEM (8), coupled with our ability to produce these samples from high-purity chains, provides a unique opportunity to understand the atomic-level structures and detect atomic structural differences in crystal packing resulting from the different sequences. A noncharged hydrophilic Nte block was chosen to eliminate interference of charged molecules with the electron beam.

### Self-Assembly of Diblock Copolypeptoids into Crystalline Nanosheets.

Diblock copolypeptoid nanosheets were formed in aqueous/tetrahydrofuran (THF) solution by an evaporation method to drive the crystallization of the aromatic hydrophobic blocks. Interestingly, all of the polypeptoids except Nte_4_-N4NO_2_pe_6_ (**6**) formed high-aspect-ratio nanosheets with nanometer-scale thickness (≤4 nm; Tables 1 and 2), as evidenced by TEM and AFM analysis (Fig. 1 *B*–*E* and *SI Appendix*, Figs. S21–S26). The most electron-withdrawing nitro group [Hammett parameter σ_p_ = 0.75; σ_p_ is a measure of inductive electron withdrawal or donation by the substituent at the *para* position of benzene (17)] in compound **6** somehow disfavors its packing into ordered nanosheets. Sharp diffraction peaks from X-ray scattering (*Temperature-Dependent Wide-Angle X-Ray Scattering Analysis*) and endothermic thermal transitions from differential scanning calorimetry (DSC) analysis (*Nanosheet Calorimetry*) demonstrate that all of the polypeptoid nanosheets are highly crystalline. As expected, the Nte_4_-N4NO_2_pe_6_ (**6**), without forming ordered nanosheet structures, showed broad X-ray scattering peaks and no melting transition.

**Table 1.** Characterization data of polypeptoid nanosheets 1, 4, 7, and 9

Peptoid sheet nomenclature	V shape packing of sheets*		T_m_, °C		Nonbonded internal energy of sheets, kcal/mol^§^	*c* spacing, Å^¶^		*a* spacing, Å^#^		Thickness, nm^‖^	
	Parallel, %	Antiparallel, %	Sheet solution^†^	Dry sheets^‡^		Cryo-TEM	WAXS	Cryo-TEM	WAXS	XRD	AFM
**1** Nte_4_-Npe_6_	100	0	92	93	−2,183 ± 101	16.2	16.2	4.5	4.5	3.9	3.2 ± 0.2
**4** Nte_4_-N4Brpe_6_	0	100	—**	145	−5,334 ± 105	18.2	18.4	4.5	4.5	3.4	2.6 ± 0.1
**7** Nte_4_-N4mpe_6_	60	40	—**	152	−6,688 ± 102^††^	18.2	18.1	4.5	4.5	3.4	2.7 ± 0.1
**9** Nte_4_-(N4BrpeNpe)_3_	100	0	99	96	−3,478 ± 103	17.2	17.0	4.5	4.5	3.8	3.0 ± 0.2

* Percentage of parallel and antiparallel V shape packing along the *c* direction in sheets obtained from cryo-TEM imaging.

^†^ Melting temperature of sheets in solutions measured by nano-DSC.

^‡^ Melting temperature of dry sheets measured by DSC.

^§^ The nonbounded internal energy of sheets which is the sum of electrostatic and van der Waals contribution were obtained from MD simulation.

^¶^ *c* spacing, the distance of adjacent backbones along the *c* direction of vitrified hydrated sheets and dry sheets, measured by cryo-TEM and WAXS, respectively.

^#^ *a* spacing, the distance of adjacent backbones along the *a* direction of vitrified hydrated sheets and dry sheets, measured by cryo-TEM and WAXS, respectively.

^‖^ Thickness of dry sheets measured by XRD and AFM. The SD of the thickness measured by AFM is obtained from at least 10 sheets.

** Melting temperature is not observed within the instrument temperature range.

^††^ The nonbounded internal energy of sheet **7** is from the model of antiparallel V. The nonbounded internal energy between parallel and antiparallel V packing is similar.

**Table 2.** Characterization data of polypeptoid nanosheets 2, 3, 5, 6, 8, and 10

Peptoid sheet nomenclature	T_m_, °C*	*c* spacing, Å^†^	*a* spacing, Å^‡^	Thickness, nm^§^	
				XRD	AFM
**2** Nte_4_-N4Fpe_6_	109	16.4	4.5	3.9	3.3 ± 0.2
**3** Nte_4_-N4Clpe_6_	118	17.7	4.5	3.7	3.1 ± 0.2
**5** Nte_6_-N4Ipe_6_	172	19.0	4.5	4.0	2.9 ± 0.2
**6** Nte_4_-N4NO_2_pe_6_	—	—	—	—	—
**8** Nte_4_-N4OMepe_6_	128	17.9	4.5	3.9	3.0 ± 0.2
**10** Nte_4_-N3NBrpe_6_	99	17.8	4.5	4.0	3.1 ± 0.2

* Melting temperature of dry sheets measured by DSC.

^†^ *c* spacing, the distance of adjacent backbones along the *c* direction of dry sheets, measured by XRD.

^‡^ *a* spacing, the distance of adjacent backbones along the *a* direction of dry sheets, measured by XRD.

^§^ Thickness of dry sheets measured by XRD and AFM. The SD of the thickness measured by AFM is obtained from at least 10 sheets.

### Cryo-TEM Imaging of Nanosheets.

All of the nanosheets adopt the same rectangular crystal lattice packing with polypeptoid chains packed in an antiparallel orientation along the *c* direction and parallel along the *a* direction (Fig. 1*F*), as evidenced by the universal *a* spacing (4.5 Å) and varied *c* spacing dependent on the atomic size of the aromatic substituents obtained from X-ray scattering measurement of the nanosheets. The *c* spacing gradually increased from 16.4 Å to 19.0 Å as the *para* substitution was changed from fluorine to iodine (Table 2). In this study, 4 nanosheets (**1**, **4**, **7**, and **9**) were chosen to further investigate their packing structures at atomic level. Nte_4_-Npe_6_ (**1**) with hydrogen as a *para* substituent is the parent compound. The Nte_4_-N4Brpe_6_ (**4**) with electron-dense bromine atoms was chosen to provide increased contrast in low-dose cryo-TEM micrographs for atom localization. The electron-withdrawing bromine atoms at the *para* positions (σ_p_ = 0.27) (17) also change the electron distribution of the phenylethyl side chains. Nte_4_-N4mpe_6_ (**7**) is similarly sized to the bromine-substituted compound but contains slightly electron-donating methyl groups at the *para* position (σ_p_ = −0.19) (17) and was included as a counterpart to **4**. Polypeptoid crystals have been previously shown to form a rectangular lattice (Fig. 1*F*) (7, 18). We therefore designed Nte_4_-(N4BrpeNpe)_3_ (**9**) to have an asymmetric side-chain substitution pattern, where one face of the molecule contains phenylethyl and the other face has 4-bromophenylethyl side chains, in order to disrupt the substitution symmetry of the polypeptoid chains and see its impact on the crystal packing.

**Fig. 2.**
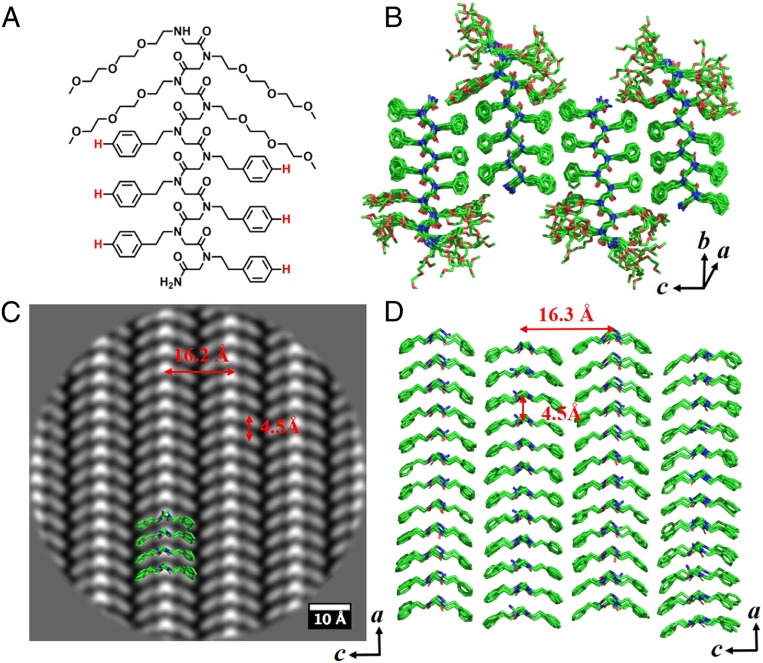
Nanosheet assembled from Nte_4_-Npe_6_ (**1**). (*A*) Chemical structure of Nte_4_-Npe_6_ (**1**). (*B*) Molecular model of sheet **1**. The molecules are packed antiparallel along *c* direction and parallel along *a* direction. (*C*) Cryo-TEM image of sheet **1** from *b* direction (top view) showing parallel V shapes along *c* direction. (*D*) Top view of the hydrophobic domain in *B* from *b* direction showing parallel V shapes along *c* direction. The structure is overlapped with cryo-TEM image shown in *C*.

**Fig. 3.**
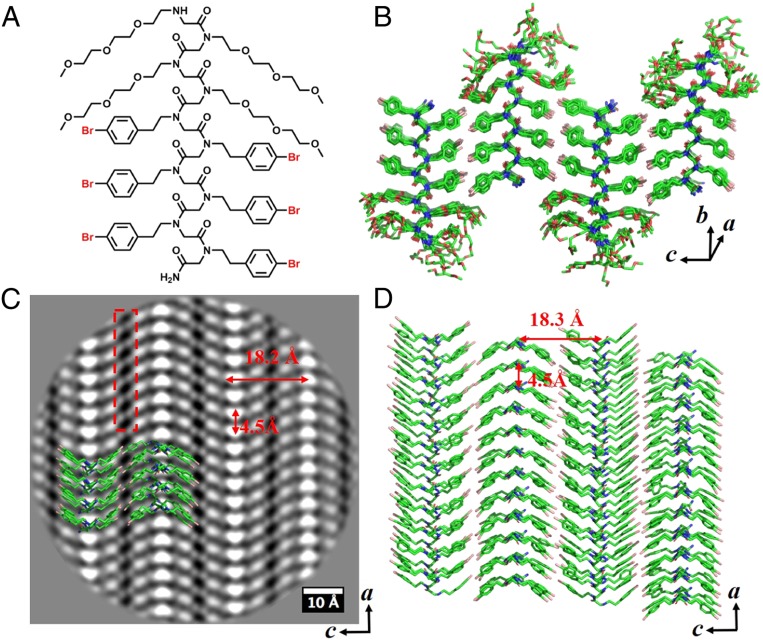
Nanosheet assembled from Nte_4_-N4Brpe_6_ (**4**). (*A*) Chemical structure of Nte_4_-N4Brpe_6_ (**4**). (*B*) Molecular model of sheet **4**. The molecules are packed antiparallel along *c* direction and parallel along *a* direction. (*C*) Cryo-TEM image of sheet **4** from *b* direction (top view) showing antiparallel V shapes along the *c* direction. The Br atoms show a tip-to-tip packing (red box). (*D*) Top view of the hydrophobic domain in *B* from *b* direction showing antiparallel V shapes along the *c* direction. The structure is overlapped with cryo-TEM image shown in *C*.

**Fig. 4.**
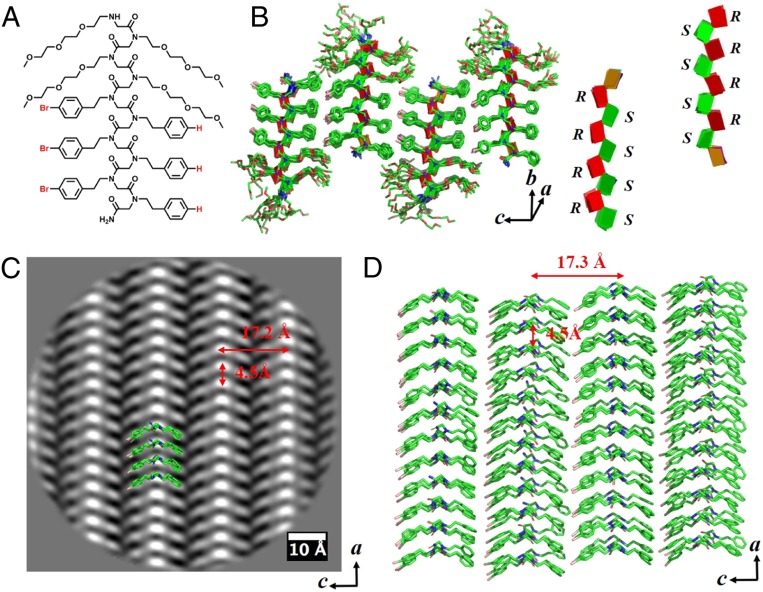
Nanosheet assembled from Nte_4_-(N4BrpeNpe)_3_ (**9**). (*A*) Chemical structure of Nte_4_-(N4BrpeNpe)_3_ (**9**). (*B*) Molecular model of sheet **9**. The molecules are packed antiparallel along *c* direction and parallel along *a* direction. The green and red planes show the opposite chirality of adjacent backbones. (*C*) Cryo-TEM image of sheet **9** from *b* direction (top view) showing parallel V shapes along the *c* direction. (*D*) Top view of the hydrophobic domain in *B* from *b* direction showing parallel V shapes along the *c* direction. The structure is overlapped with cryo-TEM image shown in *C*.

We performed low-dose cryo-TEM imaging on the vitrified hydrated crystalline nanosheets **1, 4, 7**, and **9** and the low-dose micrographs were further analyzed by sorting and averaging the small section of micrographs comprising unit cells using the protocol reported in our previous study (8). The parameters and contrast transfer function (CTF) corrections of low-dose micrographs that were used for processing can be found in *SI Appendix*, Figs. S27 and S28 and Table S2. The averaged images of nanosheets are shown in Figs. 2*C*, 3*C*, and 4*C* and *SI Appendix*, Fig. S30. The corresponding Fourier transforms (FFTs) are shown in *SI Appendix*, Figs. S29 and S30. The signal-to-noise ratio in the averaged images is significantly enhanced compared to that in original low-dose micrographs. The reflections at 1.5 Å along the *a* direction can be clearly observed. In addition, the CTF corrections of low-dose micrographs suggest the presence of detectable Thon rings in FFTs up to 1.4 Å. These results indicate the presence of reliable image phase information in the averaged images (*SI Appendix*, Fig. S28 and Table S2). In addition, at least 3 micrographs with different defocus values for each sample were used for CTF compensation during processing (see defocus values in *SI Appendix*, Table S2). The images shown in Figs. 2*C*, 3*C*, and 4*C* and *SI Appendix*, Fig. S30 are the projection views from the top view of the untilted nanosheets (*b* direction). Cryo-TEM imaging of the sheets revealed that the viewing angle is aligned with polypeptoid chains in the *b* direction, which are oriented perpendicular to the plane of the sheet, as evidenced by the projection of superimposed bromine atoms at the side chains shown in averaged images. The V shape shown is the projection of a single peptoid chain along the *b* direction. The bright dots lining up along the apex of a V-shaped structure and the arms extending out from both sides of the bright lines are the projections of backbones and side chains from the top view of the sheets, respectively. Interestingly, the dihedral angles of V shapes in the 4 sheets are similar (∼104°). Also, all of the sheets in this study show a universal *a* spacing (∼4.5 Å), which is the adjacent backbone distance along the *a* direction and is consistent with our previous study (18). The *c* spacing, the distance between backbones in adjacent rows along the *c* direction (adjacent vertical bright lines in Figs. 2*C*, 3*C*, and 4*C* and *SI Appendix*, Fig. S30), increased with increasing size of the aromatic side chains: (sheet **1**, 16.2 Å) < (sheet **9**, 17.2 Å) < (sheet **4**, 18.2 Å) = (sheet **7**, 18.2 Å).

The chosen crystalline nanosheets (except sheet **7**) exhibit homogeneity at atomic scale in terms of the presence of crystal motif, as shown in the unit cell distribution maps (*SI Appendix*, Figs. S31 and S32). Fig. 2*C* shows that the polypeptoid molecules with phenylethyl side chains in sheet **1** arranges into parallel V-shaped rows along the *c* direction. In sheet **4**, the bromine atoms (3 atoms superposed in column) at the *para* position of the phenylethyl side chains are clearly observed, showing a tip-to-tip packing from the top view of the sheet (Fig. 3*C*). It is remarkable to localize distinct atoms in position space in polymer crystals, which is inaccessible by conventional scattering technique. Interestingly, the V-shaped structure, which is the projection of individual polypeptoid chain from the top view of the nanosheets, is packed in different orientation (parallel vs. antiparallel) along the *c* direction depending on the *para* substitution of the phenylethyl side chains: sheet **1** bearing phenylethyl side chains showed all parallel V-shaped packing while sheet **4** bearing 4-bromophenylethyl side chains exhibited all antiparallel V-shaped packing (Figs. 2*C* and 3*C*).

We asked if the all antiparallel V-shaped packing in sheet **4** is due to the electron-withdrawing character of *para* Br atoms (σ_p_ = 0.27) by synthesizing Nte_4_-N4mpe_6_ (sheet **7**), which has similarly sized but slightly electron-donating methyl groups at the *para* position (σ_p_ = −0.19) (17). Surprisingly, in contrast to sheets **1** and **4**, sheet **7** exhibited about 60% antiparallel V shapes and 40% parallel V shapes, initially suggesting that the electron-withdrawing character of Br is not the primary contributor to the antiparallel V packing motif. The heterogeneity in sheet **7** is more prevalent along the *c* direction than the *a* direction, as shown in the distribution map (*SI Appendix*, Fig. S32), suggesting that polypeptoid chains are more likely to flip in the *c* direction during self-assembly.

The nanosheets with phenylethyl and 4-bromophenylethyl side chains exhibited all parallel and antiparallel V shapes, respectively. This motivated us to design the Nte_4_-(N4BrpeNpe)_3_ (sheet **9**) with an asymmetric pattern of alternating phenylethyl and 4-bromophenylethyl side chains. Surprisingly, sheet **9** adopted all parallel V shapes and the phenylethyl side chains are packed against 4-bromophenylethyl side chains. However, the packing between the shorter phenylethyl side chain and the longer 4-bromophenylethyl side chain is clearly distinguishable in the cryo-TEM image (Fig. 4*C*). Moreover, only one *c* spacing of 17.2 Å is observed in sheet **9**, which is intermediate between the *c* spacings of sheet **1** (16.2 Å in Fig. 2*C*) and sheet **4** (18.2 Å in Fig. 3*C*). The difference of spacing is caused by different van der Waals radius of bromine (∼1.85 Å) and hydrogen atoms (1.2 Å).

The different orientations of V shapes (parallel vs. antiparallel) is the result of multiple weak interactions between the interfaces along the *c* direction, making it difficult to predict. However, our studies indicated that there is not clearly one dominant factor, steric or electrostatic character of *para* substituents, for the different orientations of V shapes. Further investigation probing the origin of the packing is underway.

### MD Simulations.

We used the information derived from the atomic-scale resolution averaged images to perform MD simulations to get a better understanding of the nanosheet structures at an atomic level. MD simulations were conducted via the NAMD package (19) together with MFTOID force field (20) and the resultant models are shown in Figs. 2–4. Initial coordinates for MD simulations were generated with all backbones in the *cis*-amide conformation as suggested in recent studies (18, 21). The backbone Φ and Ψ angles of the Cα alternated between Z*_Rc_* (∼90, ∼150) and Z*_Sc_* (∼−90, ∼−150) (22). Peptoid chains were arranged in blocks of 12 stacked parallel in the *a* dimension, with these blocks arranged ether parallel or antiparallel to one another in the *c* dimension to form a periodic monolayer configuration. The models generated are well-matched with the averaged images, as shown in Figs. 2*C*, 3*C*, and 4*C* of their overlapped structures. Remarkably, the polypeptoid backbone fold itself is identical in all 4 sheet lattices (sheets **1, 4, 7**, and **9**), as shown by their superimposable structures (*SI Appendix*, Fig. S34), suggesting the capability of atomic engineering of the sheet structure predictively. Interestingly, the molecular simulation required racemic pairing of backbones with opposite chirality in sheet **9** bearing alternating phenylethyl and 4-bromophenylethyl side chains (as seen in Fig. 4*B*). Many studies have shown that racemic crystals of oligoamides (23, 24), peptides (25, 26), and proteins (27, 28) grow much more readily than the enantiomeric pure crystals. There is also a genuine tendency that racemic crystals are more stable and denser than their chiral counterparts (29). Our findings in sheet **9** are consistent with those studies. In addition, the relaxed molecules and their potential projection maps exhibit the same features as those observed in the averaged cryo-TEM images (*SI Appendix*, Fig. S33).

### Nanosheet Calorimetry.

**Fig. 5.**
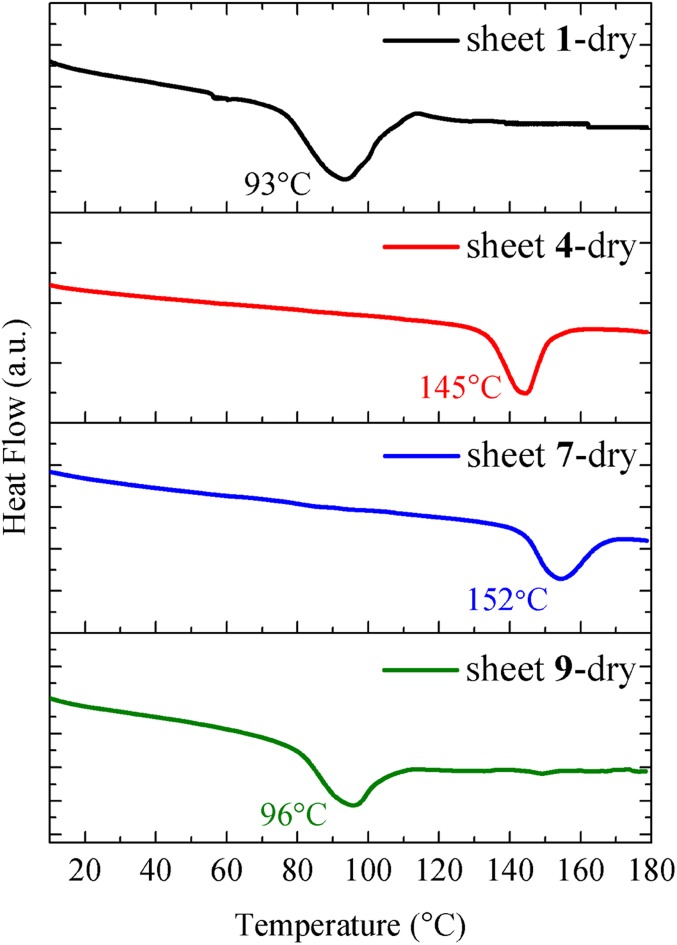
DSC measurements for dry sheets **1**, **4**, **7**, and **9**.

To investigate the effect of *para* substitution of phenylethyl side chains on the thermal properties of the nanosheets, DSC measurements were conducted on nanosheets both in solution and in the dry state. As the size of the *para* substituents increased from H to F, Cl, Br, and I, the melting temperature of the dry nanosheets gradually increased from 93 °C to 172 °C due to the enhanced van der Waals interaction between molecules (Tables 1 and 2). Surprisingly, sheets **4** and **7** with the *para* positions of phenylethyl groups fully substituted with -Br and -CH_3_, respectively, exhibited a much higher melting temperatures (∼150 °C) than those of sheets **1** and **9** (∼95 °C) with either no or partial *para* substitution (Table 1 and Fig. 5). We attribute this to the different packing geometry of side chains between rows. Since the overall molecular conformation and face-to-face *a* direction stacking is identical among all 4 molecules (*MD Simulations*), one of the primary differences among their lattices becomes the interface between adjacent rows in the *c* direction. The *para* substituents of adjacent rows essentially point directly at one another. Sheets **4** and **7** with fully *para*-substituted side chains had stronger interaction between rows due to the interdigitated packing between *para* substituents of the side chains. Sheets **1** and **9**, on the other hand, with either no or partial *para* substituents had a slight offset between rows along the *b* direction, resulting in a thicker sheet and less chain overlap. This effect is evidenced by the AFM and X-ray diffraction (XRD) data, showing that sheets **1** (3.2 nm) and **9** (3.0 nm) are thicker than sheets **4** (2.6 nm) and **7** (2.7 nm) (Table 1). The thickness of the sheets obtained from AFM is lower than that measured from XRD, which should be due to the different techniques used, the different interaction between the sheets and that between the sheets and the AFM substrate. Analysis of the MD simulations of these 4 lattices enabled us to compare their calculated total nonbonded internal energies (i.e., the sum of the electrostatic and van der Waals contributions). Sheets **4** and **7** with significantly higher melting temperatures (T_m_: ∼150 °C) showed much lower nonbonded internal energy (E_internal_ < 3,500 kcal/mol) than those of sheets **1** and **9** (T_m_: ∼90 °C and E_internal_ > 5,300 kcal/mol) (Table1), suggesting that the structures of sheets **4** and **7** are more stable than those of sheets **1** and **9**.

### Temperature-Dependent Wide-Angle X-Ray Scattering Analysis.

**Fig. 6.**
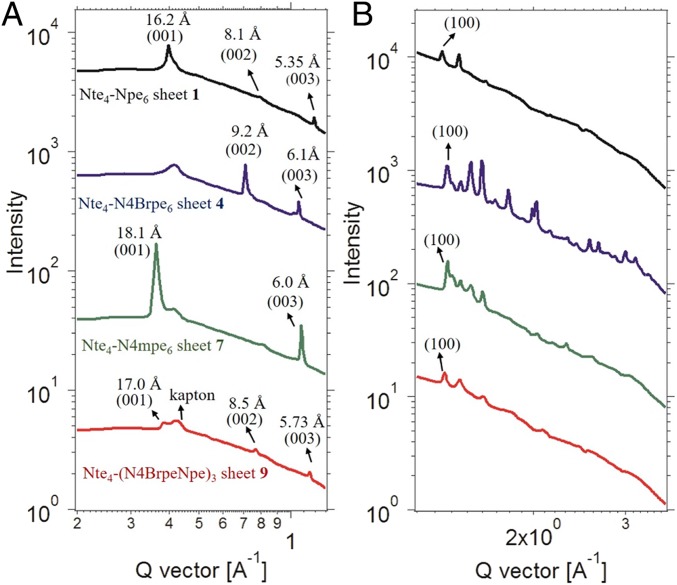
WAXS measurements of dry sheets **1**, **4**, **7**, and **9** at room temperature. (*A*) WAXS measurements showing the peaks corresponding to the *c* dimension. The peak at *q* = 0.4 Å^−1^ is from the Kapton windows. (*B*) WAXS measurements showing the peaks corresponding to the *a* dimension.

To further investigate the crystal structures of the sheets, wide-angle X-ray scattering (WAXS) measurements were conducted at increasing temperatures. Fig. 6 shows the WAXS intensity of dry sheets versus magnitude of the scattering vector, *q*, at room temperature. The *a* (100) and *c* (001) spacings of the sheets obtained from WAXS measurements are consistent with that observed from cryo-TEM imaging, as shown in Table 1. For sheet **4** bearing the 4-bromophenylethyl side chains, the scattering peak of the *c* spacing (18.2 Å) was not observed in WAXS, XRD, and solution small-angle X-ray scattering (SAXS) measurements (Fig. 6*A* and *SI Appendix*, Figs. S35 and S38); however, higher-order scattering peaks of the *c* spacing (9.2 Å and 6.1 Å) were clearly seen in Fig. 6*A* and *SI Appendix*, Fig. S35. This is probably due to the interference of the strong scattering from the bromine atoms between the side chains. Sheets **1** and **9** containing all parallel V shapes showed different diffraction patterns in the high *q* region (*q* = 1.1 to 1.7 Å^−1^) corresponding to the *a* spacing compared to sheets **4** and **7** having antiparallel V shapes (Fig. 6*B*). This is consistent with the observation from cryo-TEM imaging showing different crystal lattices: parallel vs. antiparallel V-shaped packing.

The trend of the order–disorder temperatures of the sheets (T_ODT_: sheet **7** > sheet **4** > sheet **9** > sheet **1**; *SI Appendix*, Figs. S39–S42) is consistent with the melting temperature trend obtained from DSC measurements. The change of the scattering peaks as a function of increasing temperature for all of the sheets in this study differs from that of the aliphatic nanosheet (Ac-Ndc_9_-Nte_9_) system (30) we studied previously. The broad scattering peak characterizing a loss of order along the *a* direction with no loss of order along the *c* direction with increasing temperature—a distinct signature of the sanidic liquid crystalline phase in the Ac-Ndc_9_-Nte_9_ crystal—is not observed in the sheets in this study. In this study, the scattering peaks of the sheets from *a* and *c* spacings exhibit no discernible changes before reaching the order–disorder transition temperature, where all of the scattering peaks disappear simultaneously (*SI Appendix*, Figs. S39–S42). This suggests that the sheets in the current study are not sanidic liquid crystalline, which is probably due to the more rigid aromatic side chains compared to the aliphatic side chains in the Ac-Ndc_9_-Nte_9_ crystal. The sanidic liquid crystalline phase has generally been found in polymers with flexible alkyl side chains emanating from an extended and rigid aromatic backbone (31, 32).

## Conclusions

We present atomic-level structural tuning of a peptoid nanosheet crystal lattice. The precision of peptoid synthesis, coupled with our advances in cryo-TEM imaging to directly image individual molecules in the lattice, allow us to study the polypeptoid sheet crystals at the atomic level. Remarkably, bromine atoms superposed in columns were directly and distinctly observed in position space in the nanosheet crystal bearing 4-bromophenethyl side chains. We explored the relationship between the atomic crystal structure and the aromatic side chain substitution of the polypeptoid nanosheet crystals. Interestingly, all of the sheets exhibited the same *cis* backbone fold but different lattice packing geometry (parallel vs. antiparallel V shapes) depending on the *para* substitution of the aromatic side chains. This allows us to further engineer the crystal structures by tailoring the aromatic side chains. It is interesting that breaking the symmetry of the polypeptoid, by putting alternating phenylethyl and 4-bromophenylethyl side chains, resulted in packing of phenylethyl side chains against the 4-bromophenylethyl side chains across the interface between rows in the *c* direction. This provides a precise platform to study subtle pairwise interactions between lattice faces within a polymer crystal. Interestingly, the MD simulation of this sheet required adjacent rows to pack with alternating chirality. This is consistent with other studies showing that racemic crystals grow more readily than their enantiomeric counterparts. Nanosheets exhibiting higher melting temperatures (∼150 °C) are more stable, as evidenced by their lower thickness and lower nonbonded internal energy. Our study on the investigation of polypeptoid crystal structures at the atomic level is not only a tremendous advance in soft material imaging but also in enabling the future design of biomimetic nanomaterials with more precisely controlled structures and properties.

## Materials and Methods

Detailed experimental procedures and characterization compounds can be found in *SI Appendix*.

### Synthesis of Diblock Copolypeptoids.

All diblock copolypeptoids were synthesized using automated solid-phase submonomer synthesis on a Symphony X peptide synthesizer at a scale of 200 mg Rink amide resin (0.64 mmol/g) by adapting reported procedures (18); 40 to 50 mg of final peptoid with >95% molecular purity was obtained.

### Self-Assembly of Nanosheets.

The purified diblock copolypeptoid was dissolved in THF/H_2_O (50/50, vol/vol) at a concentration of 2 mg/mL to form a clear solution, followed by slow evaporation of the THF in the refrigerator at 4 °C. Turbid solutions containing a large amount of crystalline nanosheets were obtained after several days.

### Cryo-TEM Data Collection.

The atomic-scale low-dose cryo-TEM results reported in this paper are based on vitrified, hydrated nanosheets prepared using Vitrobot (FEI Inc.). The specimens were imaged with a Titan Krios (FEI Inc.) operated at 300 kV with a K2 Summit direct detection camera and postcolumn energy filter (slit width at 25 eV) (Gatan Inc.). Low-magnification images of nanosheets were obtained from dry specimens. Micrographs were collected on a Philips CM200 at 200 kV using a Gatan US1000 charge-coupled device camera at liquid nitrogen temperature in low-dose mode to minimize radiation damage.

### Image Processing.

Images of 2D crystals are generally not perfect due to dislocations, distortions from stress, and image distortion within the microscope, which cause high-resolution diffraction spots to be smeared out. In order to recover the high-spatial-frequency signal, a crystal unbending process was conducted on all micrographs.

### Nano-DSC Measurement of Nanosheet Solutions.

Nano-DSC measurement of aqueous nanosheet solutions was performed on a CSC Model 6100 Nano differential scanning calorimeter. The nanosheet solutions were degassed and heated from 5 °C to 100 °C at 2 °C/min under a pressure of 3 atm.

### DSC Measurement of Dry Nanosheets.

DSC measurement of dry nanosheets was performed on a TA Q200 differential scanning calorimeter. The nanosheet solutions were added to a preweighed aluminum T Zero pan and dried under vacuum. This process was repeated several times until about 2 mg of dry nanosheets was collected. The DSC pan was sealed with an aluminum T Zero lid and heated from 0 °C to 180 °C at 10 °C/min.

### AFM Imaging of Nanosheets.

Diluted nanosheet solutions were dropped onto freshly cleaved mica and dried under vacuum for AFM imaging. Ex situ (in air) AFM imaging of the nanosheets was performed on an Asylum MFP-3D atomic force microscope using tapping mode imaging with TAP 150 AL-G tips. The resonant frequency and force constant of the tips are 150 kHz and 5 N/m, respectively.

### X-Ray Scattering of Crystalline Nanosheets.

The nanosheet solutions were placed on a Kapton window (25-µm thickness) which is beneath a rubber gasket spacer (0.75 cm thickness) with a hole in the center (d = 0.25 inch) and dried under vacuum. This process was repeated several times until enough nanosheets were placed on the Kapton window. Then, another Kapton window (25-µm thickness) was placed on top of the rubber spacer before placing it in the WAXS holder. WAXS measurements on the nanosheets were performed at Advanced Light Source (ALS) beamline 7.3.3 located at Lawrence Berkeley National Labratory.

### Powder XRD.

The nanosheet solutions were pipetted onto a MiTeGen micromesh and dried under vacuum. This process was repeated several times until enough nanosheets were placed on the micromesh. Powder XRD data of the nanosheets were collected at ALS beamline 8.3.1, using multiple-wavelength anomalous diffraction and monochromatic macromolecular crystallography. Beamline has a superbend source with an energy range of 5 to 17 keV. Data were collected with the detector 350 mm from the sample.

### High-Throughput SAXS.

The nanosheet solutions were diluted with miliQ water to prepare samples for 3 different concentrations. SAXS data were collected at the SIBYLS beamline (12.3.1) at the ALS in the Lawrence Berkeley National Laboratory. The sample was collected at 11 keV (1.27 Å) X-ray beam. The SAXS data were analyzed by ScÅtter.

### MD Simulations.

Initial coordinates for MD simulations were generated with all backbones in the *cis*-amide conformation and with Φ and Ψ angles of the Cα alternated between Z*_Rc_* (∼90, ∼150) and Z*_Sc_* (∼−90, ∼−150). Monomer chains were arranged in blocks of 12 stacked parallel in the *a* dimension with these blocks arranged either parallel or antiparallel to one another in the *c* dimension to form a periodic monolayer configuration. Topology information and psf generation were performed using autopsf in VMD (33). Water molecules were added using the autosol plugin in VMD. Simulations were run in NAMD (19) with CHARMM force-field parameters similar to MFTOID (20). Simulations were run at 300 K for at least 50 ns under an isothermal-isobaric ensemble. The nonbonded internal energy of polypeptoid sheets was calculated using the molecular mechanics energy function in NAMD 2.12 (19). The statistical analysis was carried out on internal energy data for the last 20-ns MD trajectory.

## Supplementary Material

Supplementary File

Supplementary File

Supplementary File
