# Supplementary material for: Atomic-level engineering and imaging of polypeptoid crystal lattices
Source: Proc Natl Acad Sci U S A. 2019 Oct 21;116(45):22491–9. doi: 10.1073/pnas.1909992116 (PMC6842587; doi:10.1073/pnas.1909992116)
Supplement: Supplementary File [file pnas.1909992116.sapp.pdf]

Supplementary Information

Atomic-level Engineering and Imaging of Polypeptoid Crystal Lattices

Sunting Xuan[a,1], Xi Jiang[b,1], Ryan K. Spencer[c], Nan K. Li[a], David Prendergast[a], Nitash P. Balsara[b,d], and Ronald N. Zuckermann[a,2]

[a]Molecular Foundry, Lawrence Berkeley National Laboratory, Berkeley, CA 94720, United States.

[b]Materials Sciences Division, Lawrence Berkeley National Laboratory, Berkeley, CA 94720, United States.

[c]Department of Chemistry and Department of Chemical Engineering & Materials Science, University of California, Irvine, CA 92697, United States

[d]College of Chemistry, University of California, Berkeley, Berkeley, CA 94720, United States.

[1] S.X. and X. J. Contributed equally to this work.

Table of Contents

Synthesis of diblock copolypeptoids 3
Self-assembly of nanosheets 4
Cryo-TEM data collection 4
Image processing 5
Table S1. Characterization data of polypeptoids **1-10**. 6
Fig. S1. UPLC-MS trace of purified Nte_4-Npe_6 (**1**) 7
Fig. S2. UPLC-MS trace of purified Nte_4-$N4Fpe_6$ (**2**) 8
Fig. S3. UPLC-MS trace of purified Nte_4-$N4Clpe_6$ (**3**) 9
Fig. S4. UPLC-MS trace of purified Nte_4-$N4Brpe_6$ (**4**) 10
Fig. S5. UPLC-MS trace of purified Nte_6-$N4Ipe_6$ (**5**) 11
Fig. S6. UPLC-MS trace of purified Nte_4-$N4NO_2pe_6$ (**6**) 12
Fig. S7. UPLC-MS trace of purified Nte_4-$N4mpe_6$ (**7**) 13
Fig. S8. UPLC-MS trace of purified Nte_4-$N4OMepe_6$ (**8**) 14
Fig. S9. UPLC-MS trace of purified Nte_4-$(N4BrpeNpe)_3$ (**9**) 15
Fig. S10. UPLC-MS trace of purified Nte_4-$N3Brpe_6$ (**10**) 16
Fig. S11. ^1H and ^{13}C NMR spectra of Nte_4-$N4pe_6$ (**1**) in CD_2Cl_2. 17
Fig. S12. ^1H and ^{13}C NMR spectra of Nte_4-$N4Fpe_6$ (**2**) in CD_2Cl_2. 18
Fig. S13. ^1H and ^{13}C NMR spectra of Nte_4-$N4Clpe_6$ (**3**) in CD_2Cl_2. 19
Fig. S14. ^1H and ^{13}C NMR spectra of Nte_4-$N4Brpe_6$ (**4**) in CD_2Cl_2. 20

www.pnas.org/cgi/doi/10.1073/pnas.1909992116

Fig. S15. ^1H and ^{13}C NMR spectra of Nte_6-N4Ipe$_6$ (**5**) in CD_2Cl_2 and DMSO-d^6, respectively. The ^{13}C NMR was conducted in DMSO-d^6 due to limited solubility at high concentration in CD_2Cl_2. 21

Fig. S16. ^1H and ^{13}C NMR spectra of Nte_4-N4NO2pe$_6$ (**6**) in CD_2Cl_2. 22

Fig. S17. ^1H and ^{13}C NMR spectra of Nte_4-N4mpe$_6$ (**7**) in CD_2Cl_2. 23

Fig. S18. ^1H and ^{13}C NMR spectra of Nte_4-N4OMepe$_6$ (**8**) in CD_2Cl_2. 24

Fig. S19. ^1H and ^{13}C NMR spectra of Nte_4-(N4Brpe$_6$Npe)$_3$ (**9**) in CD_2Cl_2. 25

Fig. S20. ^1H and ^{13}C NMR spectra of Nte_4-N3Brpe$_6$ (**10**) in CD_2Cl_2. 26

Fig. S21. TEM images of dry sheet Nte_4-N4Brpe$_6$ (**4**) (a), Nte_4-(N4BrpeNpe)$_3$ (**9**) (b), Nte_4-N4Fpe$_6$ (**2**) (c), Nte_4-N4Clpe$_6$ (**3**) (d), Nte_6-N4Ipe$_6$ (**5**) (e), Nte_4-N4NO_2pe$_6$ (**6)** (f), and Nte_4-N4OMepe$_6$ (**8**) (g) and Nte_6-N3Brpe$_6$ (**10**). 27

Fig. S22. AFM images of dry sheet Nte_4-Npe$_6$ (**1**): (a, b) height image; (c, d) corresponding phase image. The inset graph in (b) is the thickness profile of the sheets. 27

Fig. S23. AFM images of dry sheet Nte_4-N4Brpe$_6$ (**4**): (a, b) height images; (c, d) corresponding phase images. The inset graph in (b) is the thickness profile of the sheet. 28

Fig. S24. AFM images of dry sheet Nte_4-N4mpe$_6$ (**7**): (a, b) height images; (c, d) corresponding phase images. The inset graph in (b) is the thickness profile of the sheet. 28

Fig. S25. AFM images of dry sheet Nte_4-(N4BrpeNpe)$_3$ (**9**): (a, b) height images; (c, d) corresponding phase images. The inset graph in (b) is the thickness profile of the sheets. 29

Fig. S26. AFM images of dry sheet Nte_4-N4Fpe$_6$ (**2**) (a), Nte_4-N4Clpe$_6$ (**3**) (b), Nte_6-N4Ipe$_6$ (**5**) (c), Nte_4-N4OMepe$_6$ (**8**) (d), and Nte_4-N3Brpe$_6$ (**10**) (e). 29

Fig. S27. Low-dose cryo-TEM micrographs of nanosheets 30

Fig. S28. CTF estimation of low-dose cryo-TEM micrographs 30

Fig. S29. FFTs of averaged images shown in Figure 2c, Figure 3c and Figure 4c 31

Fig. S30. Averaged images of sheet Nte_4-N4mpe$_6$ (**7**) showing both anti-parallel (a) and parallel (b) V shapes. (c, d) FFTs of averaged images shown in (a) and (b), respectively. 31

Fig. S31. Distribution maps of motifs in the nanosheets 32

Fig. S32. Crystal motifs and distribution map in nanosheet Nte_4-N4mpe$_6$ (**7**) 32

Fig. S33. Comparisons between simulations and cryo-TEM images 33

Table S2. Summary of properties of micrographs used in the analysis 34

Fig. S34. Overlapped peptoid chains taken from the MD sheet models showing the same backbone fold 34

Fig. S35. XRD measurements of dry sheets **1**, **4**, **7** and **9**. 35

Fig. S36. XRD measurements of dry sheets **2** (a), **3** (b), **5** (c), **6** (d), **8** (e), **10** (f). 35

Fig. S37. NanoDSC measurements of sheets Nte$_4$-Npe$_6$ (**1**) and Nte$_4$-(N4BrpeNpe)$_3$ (**9**) in water at about 4mg/mL (a) and DSC measurements of dry sheets **2**, **3**, **5**, **6**, **8**, **10**. 36

Fig. S38. SAXS measurements of sheets **1**, **4** and **9** in aqueous solutions. 36

Fig. S39. WAXS measurements of sheet Nte$_4$-Npe$_6$ (**1**) as a function of increasing temperature 37

Fig. S40. WAXS measurements of sheet Nte$_4$-N4Brpe$_6$ (**4**) as a function of increasing temperature 38

Fig. S41. WAXS measurements of dry sheet Nte$_4$-N4mpe$_6$ (**7**) as a function of increasing temperature 39

Fig. S42. WAXS measurements of dry sheet Nte$_4$-(N4BrpeNpe)$_3$ (**9**) as a function of increasing temperature 40

Reference 40

Synthesis of diblock copolypeptoids. The 2-(2-(2-methoxyethoxy)ethoxy)ethylamine was purchased from Peptide Solutions, Inc. (98% purity). Other amines including phenethylamine (99% purity), 4-bromophenethylamine (98% purity) and 4-methylphenethylamine (97% purity) were purchased from Sigma Aldrich. Rink amide resin was purchased from NOVABIOCHEM. All the other solvents and reagents described here were purchased from commercial sources and used as received. All diblock copolypeptoids were synthesized using automated solid-phase submonomer synthesis on a Symphony X peptide synthesizer at a scale of 200 mg Rink amide resin (0.64 mmol/g) by adapting reported procedures. The resin was swelled in DMF for 10min and the Fmoc group on the resin was deprotected with 20% (v/v) 4-methylpiperidine/DMF. The bromoacylation reaction was then performed with bromoacetic acid (0.8 M) and *N,N'*-diisopropylcarbodiimide (DIC, 0.8M) in DMF at room temperature for 20min. The subsequent displacement reaction with various submonomers was performed at 1 M amine concentration in *N*-methyl-2-pyrrolidone (NMP) at room temperature for 30min. The crude diblock copolypeptoids were cleaved from the resin by treating with 95% (v/v) trifluoroacetic acid (TFA) in water for 10 min at room temperature, followed by filtration and washing of the resin with DCM. The solvent was evaporated using Biotage® V-10 evaporator and the crude products were lyophilized from acetonitrile/water (1:1, v/v).

The crude peptoids were then purified by Waters reverse-phase HPLC on a C18 semipreparative column (5 µm, 250 mm × 21.2 mm, C18 Vydac column) using acetonitrile with

0.1% TFA (solvent B)/water with 0.1% TFA (solvent A) with a flow rate of 15 mL/min. The linear gradients used for the purification of Nte_4-Npe_6, Nte_4-$N4Brpe_6$, Nte_4-$(N4Brpe$-$N4pe)_3$ and Nte_4-$Nmpe_6$ are 30-70% B, 40-80% B, 40-80% B and 40-80% B over 30min, respectively. The fractions were analyzed by Waters ACQUITY reverse-phase UPLC with a ACQUITY®BEH C4 column (1.7 µm, 2.1 mm × 50 mm) connected with a Waters SQD2 mass spectrometry system using 20-80% gradient acetonitrile in water with 0.1% TFA at 0.4 mL/min over 6.8 min at 60 ^{o}C. The fractions containing pure compound were collected and the solvent was removed by Genevac evaporator, followed by lyophilization from acetonitrile/water (1:1, v/v) to obtain the fluffy white powder. 40-50 mg of final peptoid with > 95% molecular purity was obtained. All the purified polypeptoids were characterized by NMR spectroscopy on a Bruker Avance II 500 MHz at room temperature.

Self-assembly of nanosheets. The purified diblock copolypeptoid was dissolved in THF/H_2O (50/50, v/v) at a concentration of 2 mg/mL to form a clear solution, followed by slow evaporation of the THF in the refrigerator at 4 ^{o}C. Turbid solutions containing a large amount of crystalline nanosheets were obtained after several days.

Cryo-TEM data collection. The atomic-scale low-dose cryo-TEM results reported in this paper are based on vitrified, hydrated nanosheets. These specimens were prepared on C-Flat grids (Protochips Inc.) which were covered by a thin, continuous carbon film (3 nm). A 3 µl droplet of the desired nanosheet-containing aqueous solution was transferred to the grid and blotted using two filter papers. The grid was then plunged into liquid ethane to obtain vitrified hydrated specimen using Vitrobot (FEI Inc.). The specimens were imaged with a Titan Krios (FEI Inc.) operated at 300 kV with a K2 Summit direct detection camera and post-column energy filter (slit width at 25 eV) (Gatan Inc.) using defocus values from -1.0 to -0.2 µm. Dose-fractionation movies comprised 30 frames. The accumulated dose for each movie was about 21 e/Å^2. Movie frames were aligned and summed by MotionCorr2 to eliminate beam-induced motion (1). Contrast transfer function (CTF) estimations were carried out using gCTF (2). CTF correction was applied to all micrographs analyzed in this study.

Low magnification images of nanosheets were obtained from dry specimens. These specimens were prepared by depositing a 3 µl droplet of the desired nanosheet-containing aqueous solution on a continuous carbon film that was supported on a copper grid. The droplet was blotted

from the edge of the grid using a filter paper. The grid was transferred to a TEM cryo holder (914 Gatan Inc.) and micrographs were collected on a Philips CM200 at 200 kV using a Gatan US1000 CCD camera at liquid nitrogen temperature in low-dose mode to minimize radiation damage. We expect the nanosheets to be completely dry due to exposure to vacuum, first in the airlock and then in the column of the TEM.

Image processing. Images of 2D crystals are generally not perfect due to dislocations, distortions from stress and image distortion within the microscope, which cause high resolution diffraction spots to be smeared out. In order to recover the high spatial frequency signal, a crystal unbending process was conducted on all micrographs. The motion-corrected and summed low-dose micrographs were imported into 2dx, an image processing package for 2D electron crystallography (3-5). Details of the principle of crystal unbent processing can be found in Henderson et al.'s work (6, 7). Briefly, the position of each unit cell in the image is found by cross correlation with a small reference area, and a smooth function is defined for displacements from the ideal lattice. This function is then used to re-interpolate the image onto the regular lattice. The defocus values, astigmatism, and specimen tilt geometry were determined using the gCTF program and corrected after unbending.

Overlapping small square boxes (150 pixels long on each side), were extracted from the micrographs. The centers of the boxes coincide with the centers of the locations of unit cells as described in the previous work (8). The extracted boxes were sorted into image classes using the Relion software package (9, 10). Relion uses the maximum-likelihood approach for sorting the small boxes extracted from cryo-TEM micrographs (11). This approach has been proven to be particularly useful in the classification of structurally heterogeneous data from biomolecules. The intensity in the boxes is first normalized. A soft round mask is applied to the images in boxes to reduce their background noise. Reference-free class averages are obtained in a completely unsupervised manner by starting multiple references from average images of random sets of the normalized images in the extracted boxes. All images are compared to all references in all possible orientations and probability weights are calculated for each possibility instead of assigning images to one particular class or orientation. Class averages are then calculated as weighted averages over all possible assignments. The number of classes requested is set by the user: fewer boxes participate in each class with increasing number of classes. Averaging over a small number of boxes leads to noisy averages, which result in suboptimal alignment and classification. The number

of classes requested can be set within a wide range. In this study, the classification analysis suggests the presence of homogenous crystal motifs in the Nte_4-Npe_6, Nte_4-$N4Brpe_6$, and Nte_4-$(N4Brpe\text{-}N4pe)_3$ nanosheets when one class was applied to those nanosheets. However, 4 classes were applied to the Nte_4-$N4mpe_6$ nanosheets due to the presence of heterogeneity. The averaged images in 4 classes comprising similar or nearly identical motifs were separated into 3 groups.

Table S1. Characterization data of polypeptoids **1-10**.

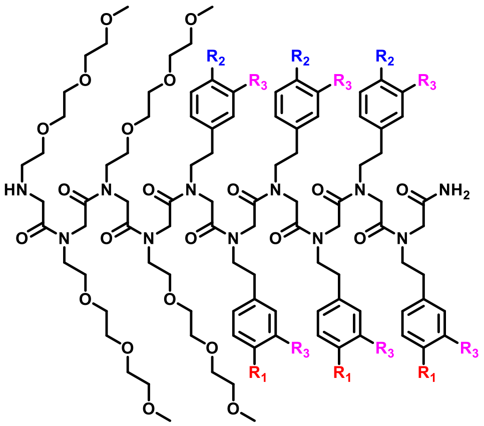

	Peptoid Nomenclature	R_1	R_2	R_3	Theoretical $[M+H]^+$	Observed $[M+H]^{+a}$	Purity[b]
**1**	Nte_4-Npe_6	H	H	H	1797	1797	97.7
**2**	Nte_4-$N4Fpe_6$	F	F	H	1906	1906	97.3
**3**	Nte_4-$N4Clpe_6$	Cl	Cl	H	2005	2005	96.0
**4**	Nte_4-$N4Brpe_6$	Br	Br	H	2272	2272	98.1
**5**	[c]Nte_6-$N4Ipe_6$	I	I	H	2961	2961	96.3
**6**	Nte_4-$N4NO_2pe_6$	NO_2	NO_2	H	2068	2068	98.5
**7**	Nte_4-$N4mpe_6$	CH_3	CH_3	H	1882	1882	98.4
**8**	Nte_4-$N4OMepe_6$	OMe	OMe	H	1978	1978	96.1
**9**	Nte_4-$(N4BrpeNpe)_3$	H	Br	H	2035	2035	97.7
**10**	Nte_4-$N3Brpe_6$	H	H	Br	2272	2272	96.3

[a] Mass was obtained from UPLC-MS. [b]Purity was determined from UPLC. [c]The Nte block was increased to 6 monomers to increase the solubility of the compound.

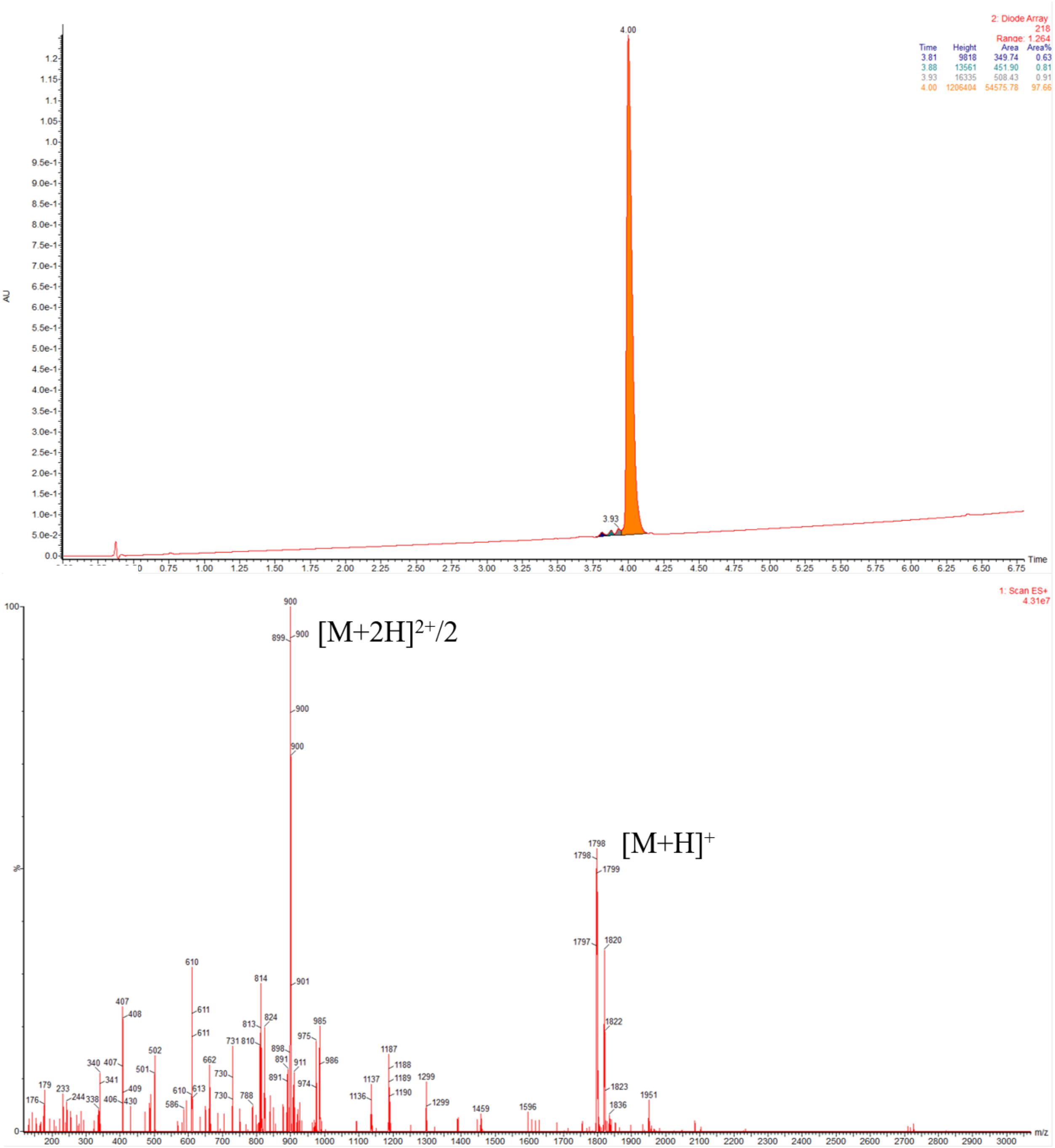

Fig. S1. UPLC-MS trace of purified Nte$_4$-Npe$_6$ (**1**) with gradient of 20-80% ACN in H_2O in 6.8 min. LC trace of **1** (top image) and mass spectrum of **1** (bottom image).

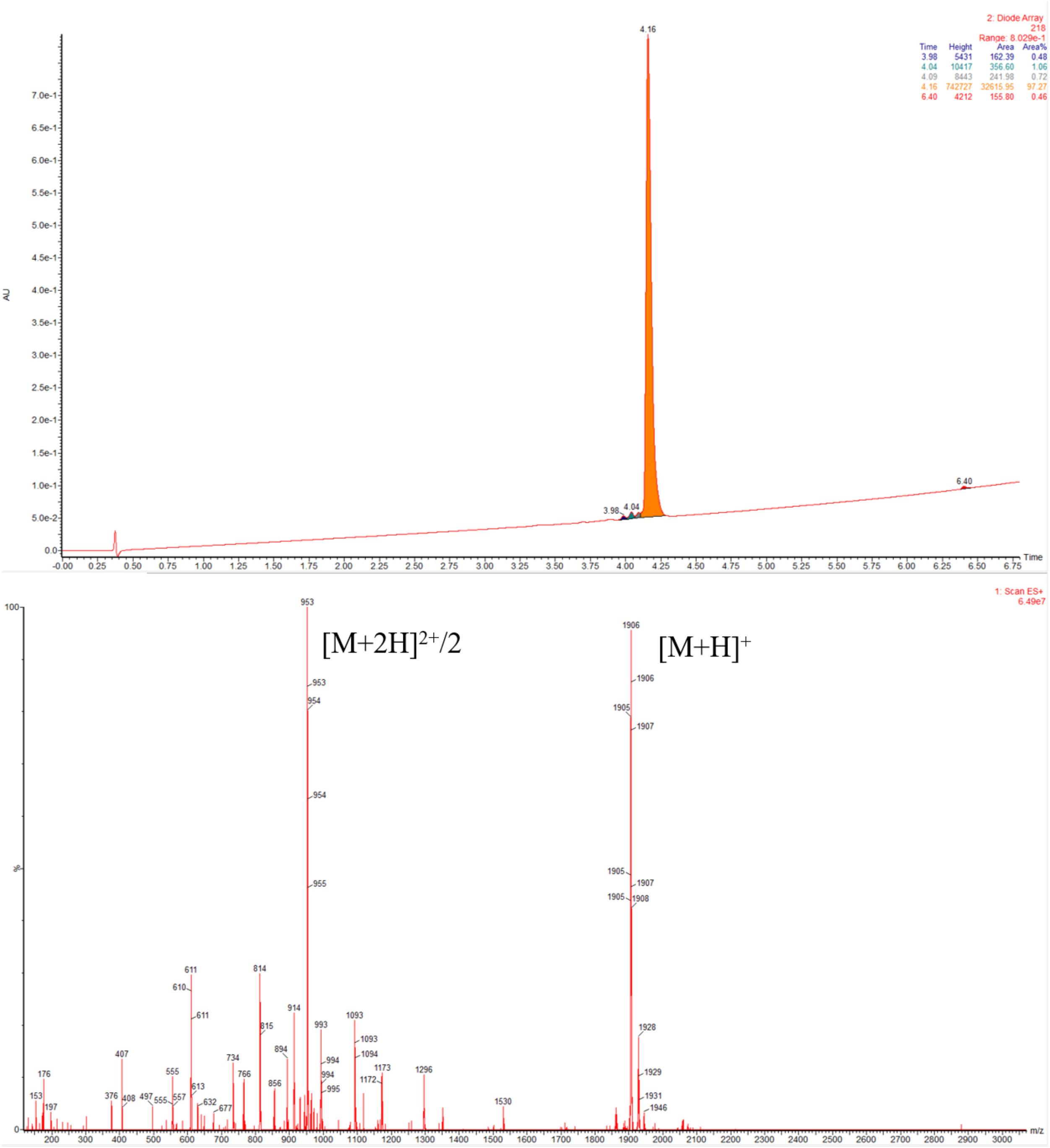

Fig. S2. UPLC-MS trace of purified Nte$_4$-N4Fpe$_6$ (**2**) with gradient of 20-80% ACN in H_2O in 6.8 min. LC trace of **2** (top image) and mass spectrum of **2** (bottom image).

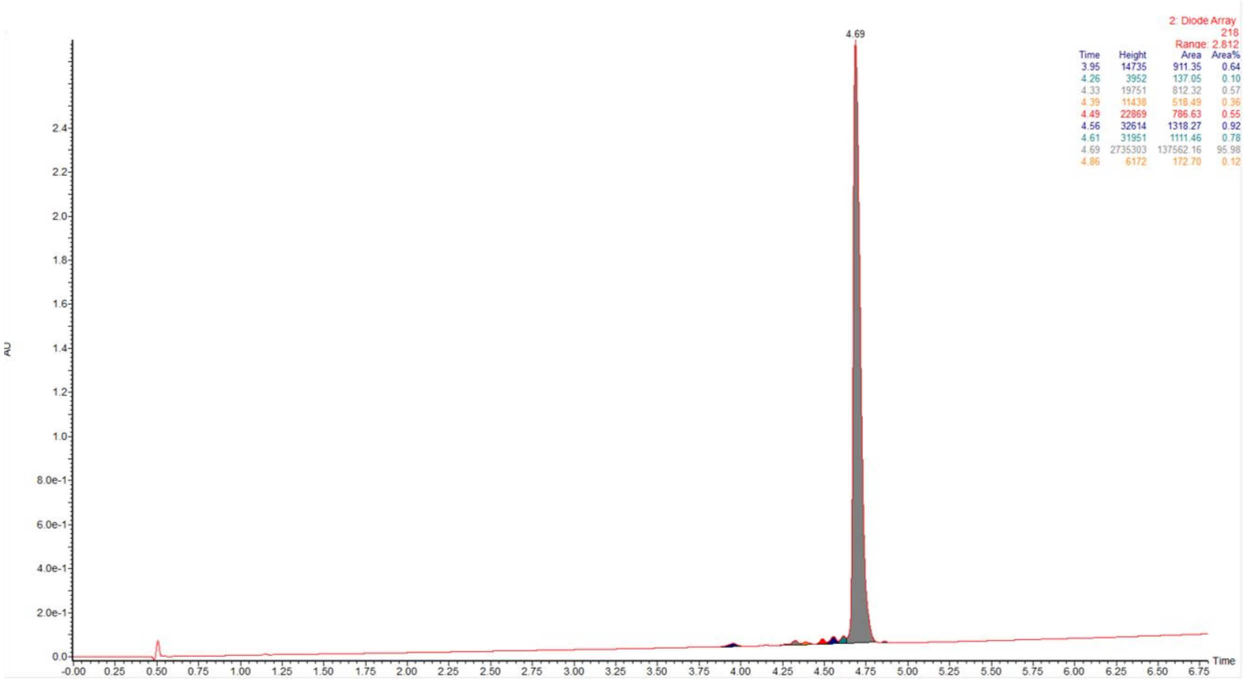

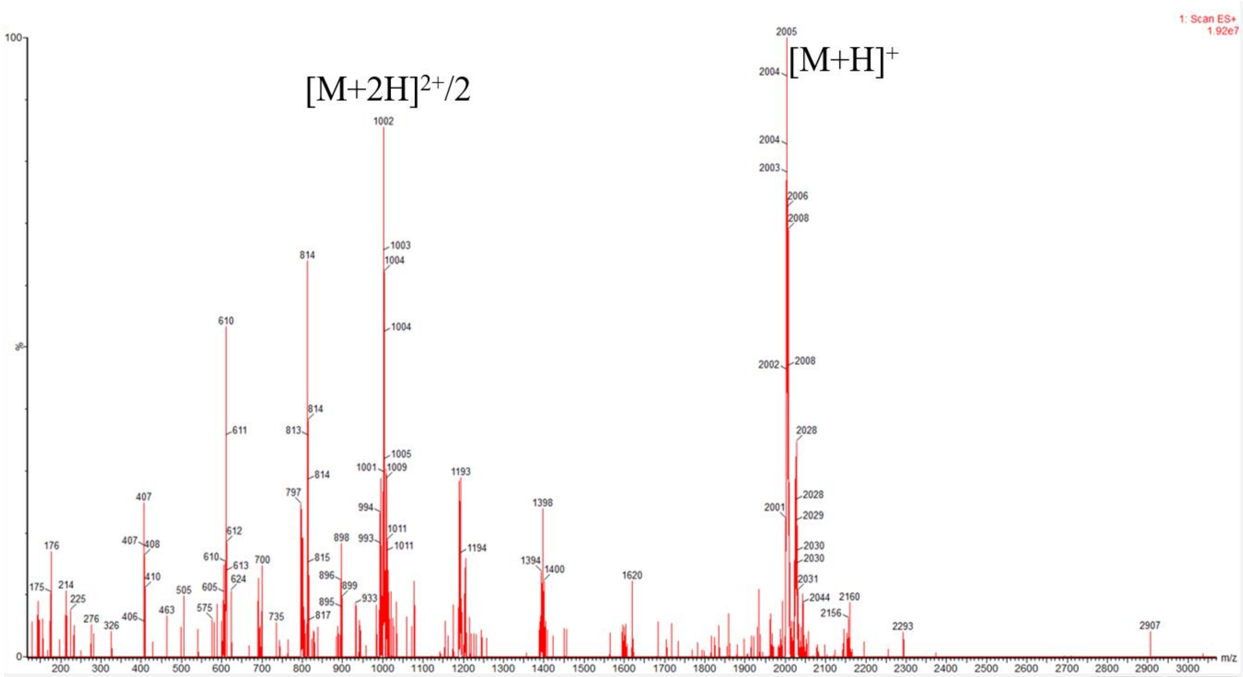

Fig. S3. UPLC-MS trace of purified Nte$_4$-N4Clpe$_6$ (**3**) with gradient of 20-80% ACN in H_2O in 6.8 min. LC trace of **3** (top image) and mass spectrum of **3** (bottom image).

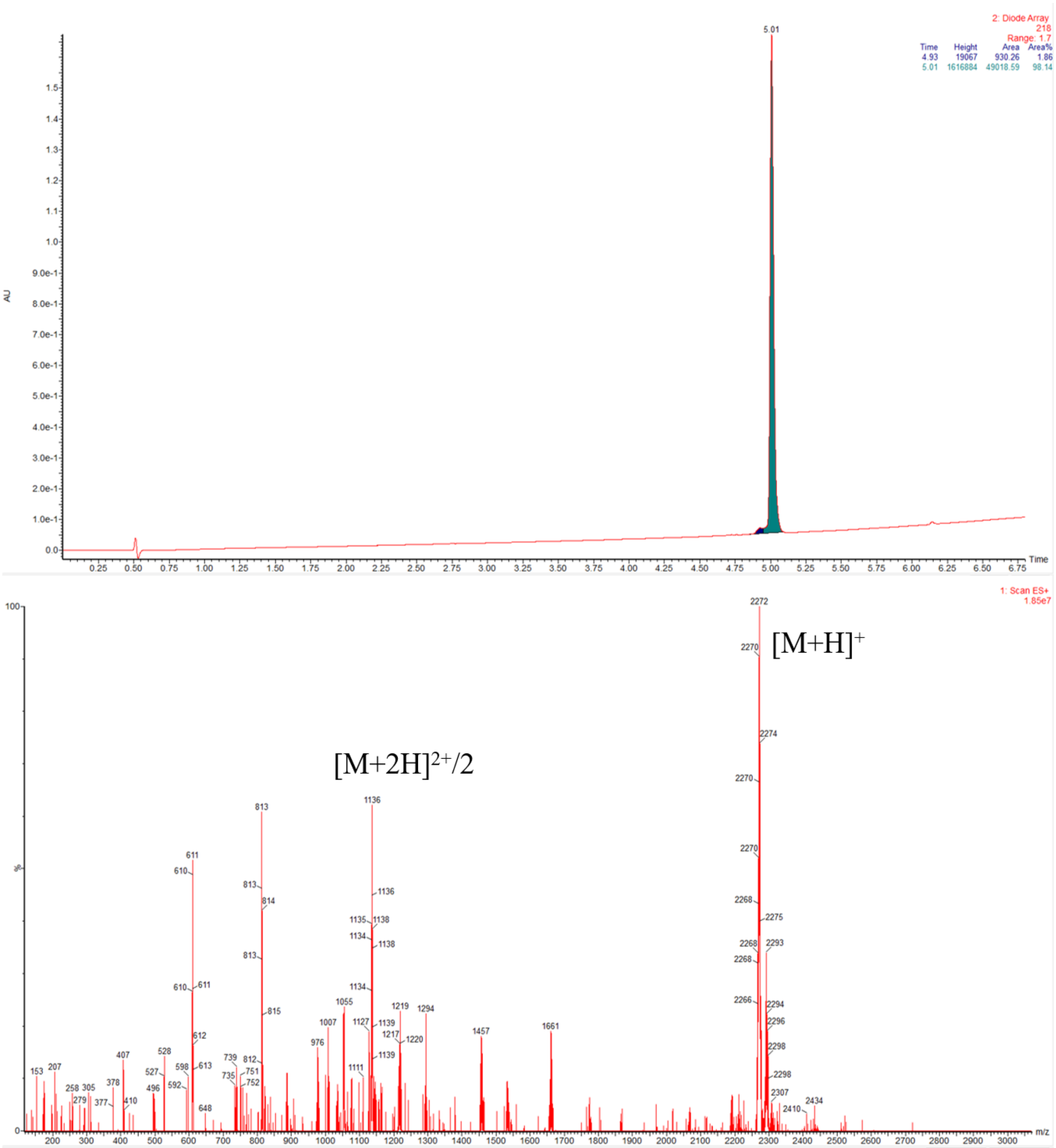

Fig. S4. UPLC-MS trace of purified Nte$_4$-N4Brpe$_6$ (**4**) with gradient of 20-80% ACN in H_2O in 6.8 min. LC trace of **4** (top image) and mass spectrum of **4** (bottom image).

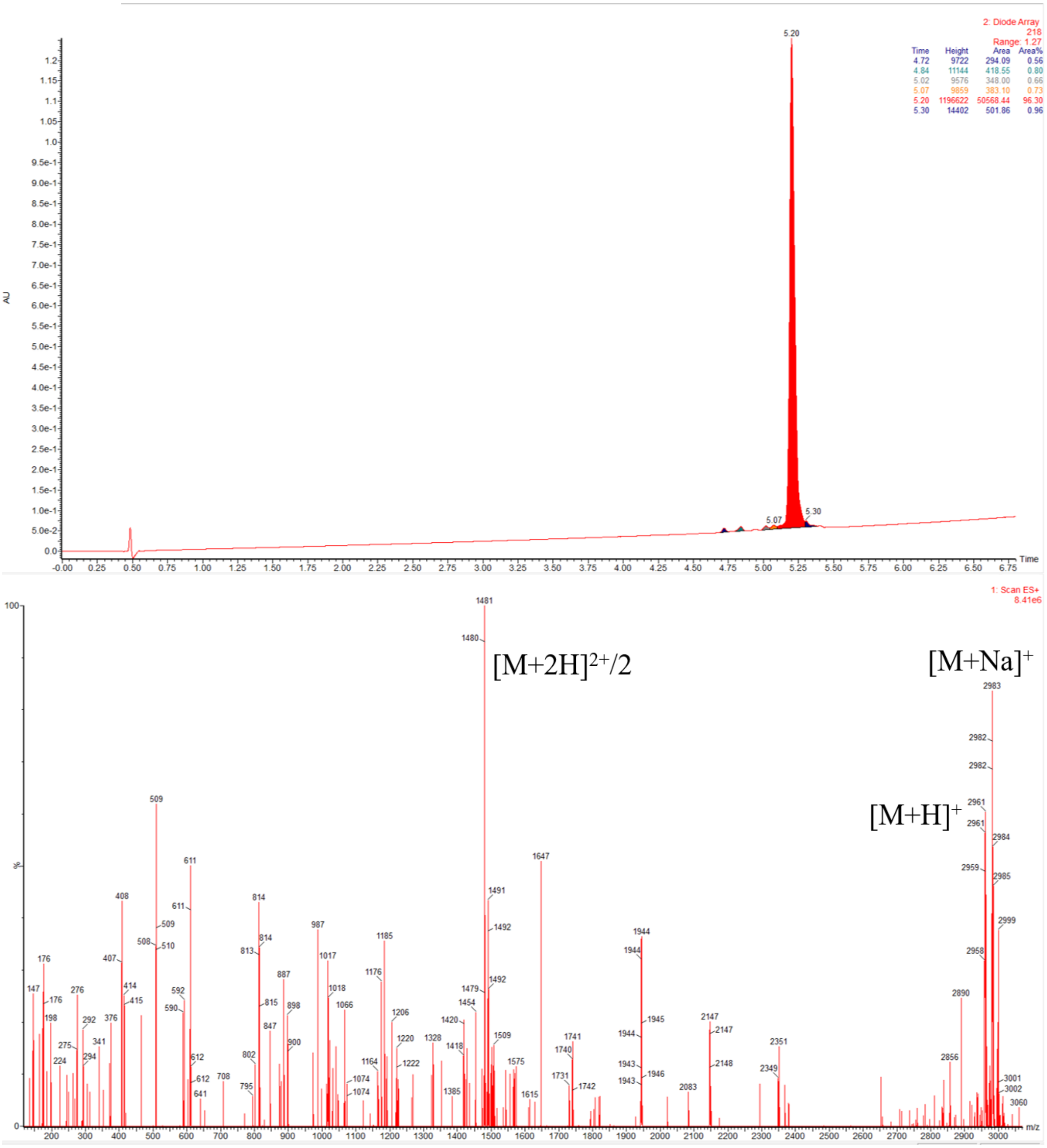

Fig. S5. UPLC-MS trace of purified Nte$_6$-N4Ipe$_6$ (**5**) with gradient of 20-80% ACN in H_2O in 6.8 min. LC trace of **7** (top image) and mass spectrum of **5** (bottom image).

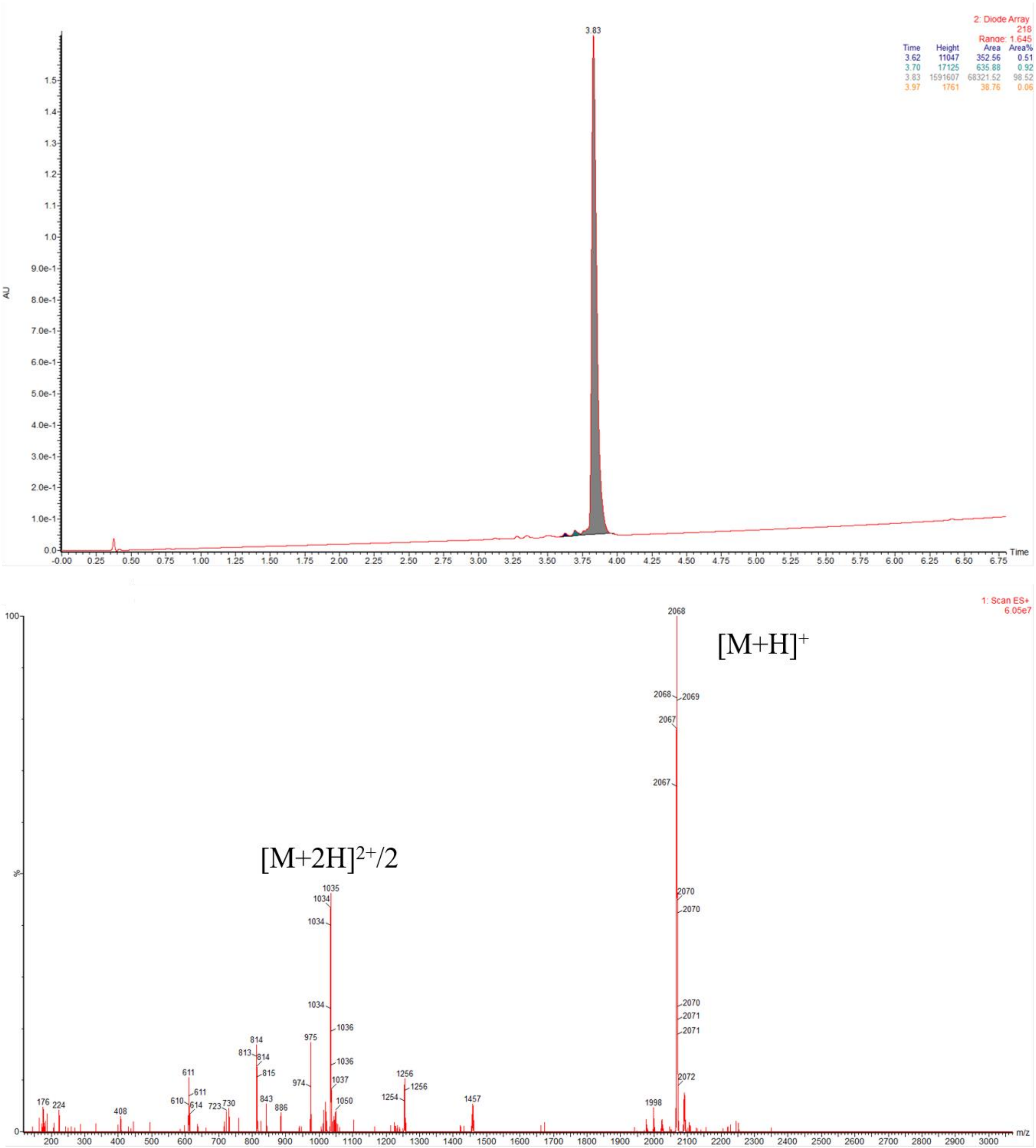

Fig. S6. UPLC-MS trace of purified Nte$_4$-N4NO$_2$pe$_6$ (**6**) with gradient of 20-80% ACN in H_2O in 6.8 min. LC trace of **6** (top image) and mass spectrum of **6** (bottom image).

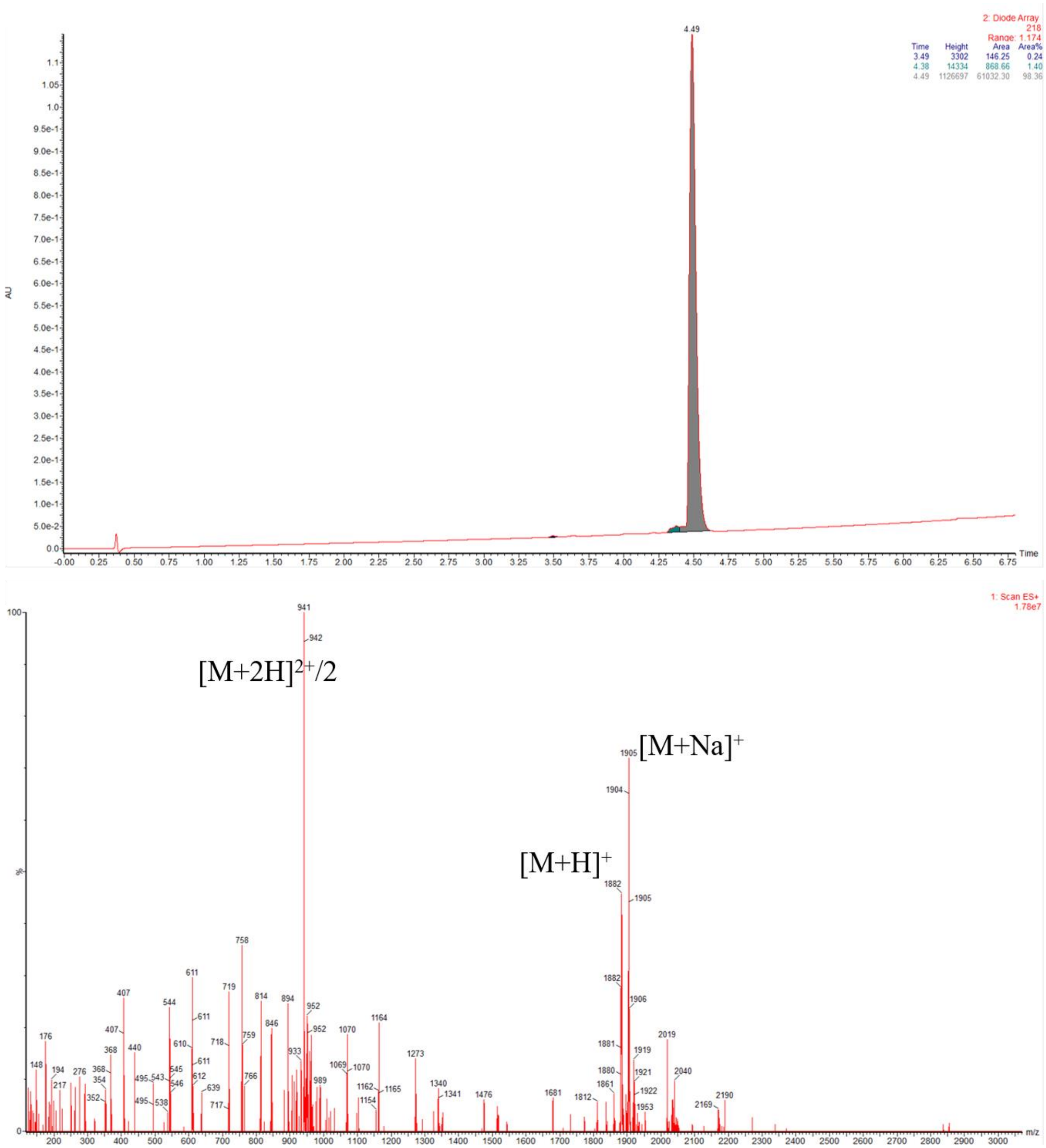

Fig. S7. UPLC-MS trace of purified Nte$_4$-N4mpe$_6$ (**7**) with gradient of 20-80% ACN in H_2O in 6.8 min. LC trace of **7** (top image) and mass spectrum of **7** (bottom image).

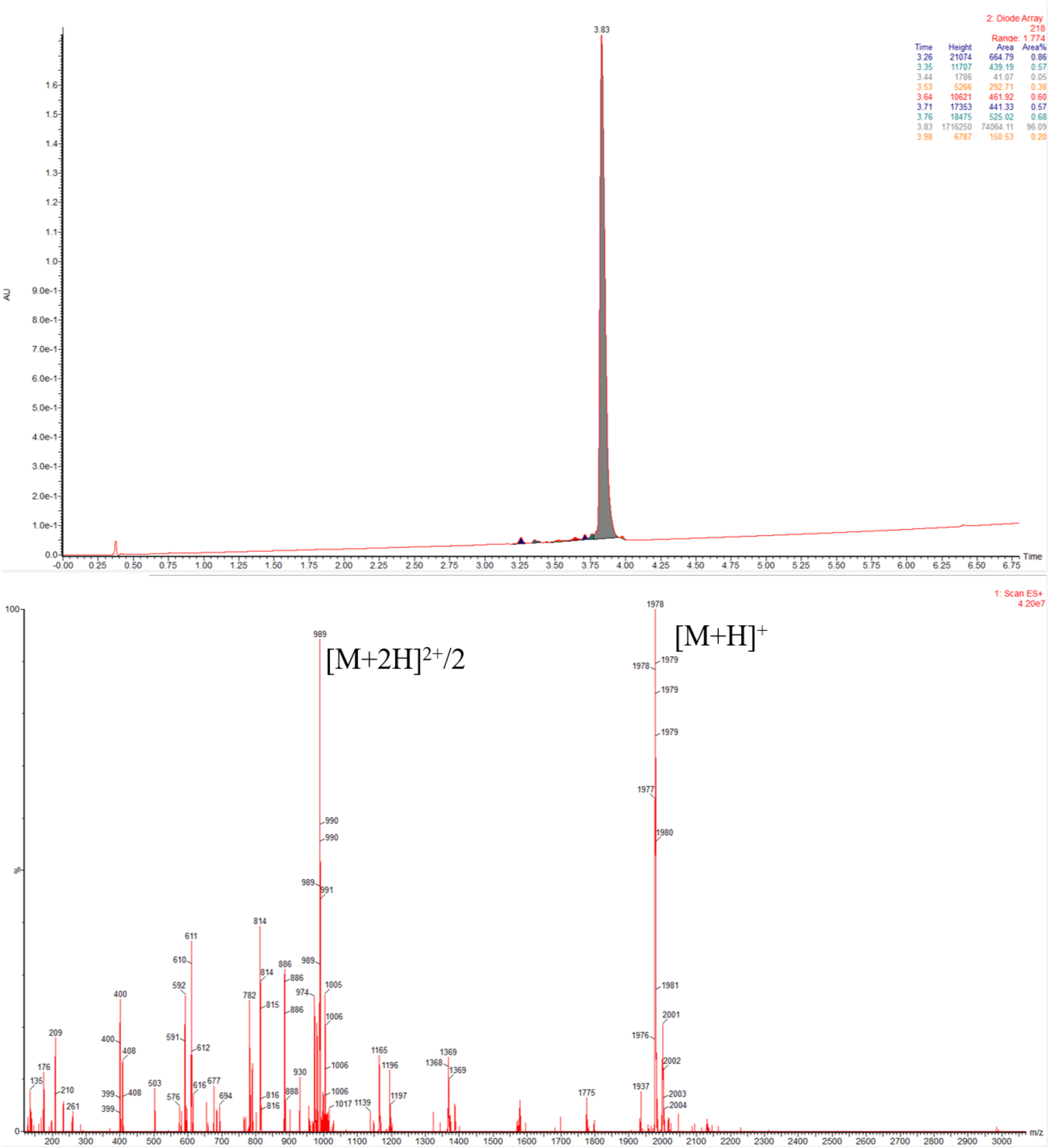

Fig.S8. UPLC-MS trace of purified Nte$_4$-N4OMepe$_6$ (**8**) with gradient of 20-80% ACN in H_2O in 6.8 min. LC trace of **8** (top image) and mass spectrum of **8** (bottom image).

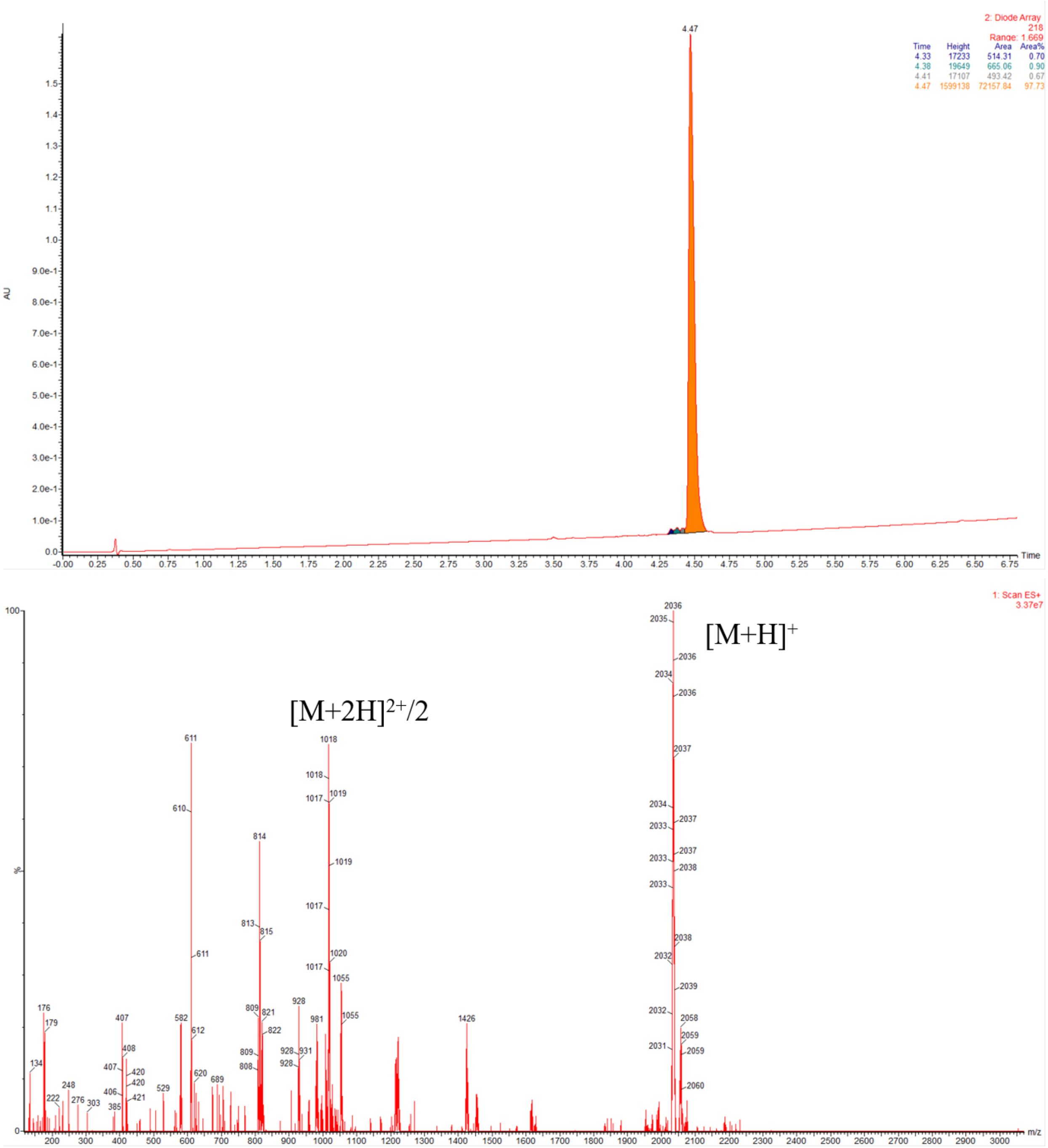

Fig. S9. UPLC-MS trace of purified Nte_4-$(N4BrpeNpe)_3$ (**9**) with gradient of 20-80% ACN in H_2O in 6.8 min. LC trace of **9** (top image) and mass spectrum of **9** (bottom image).

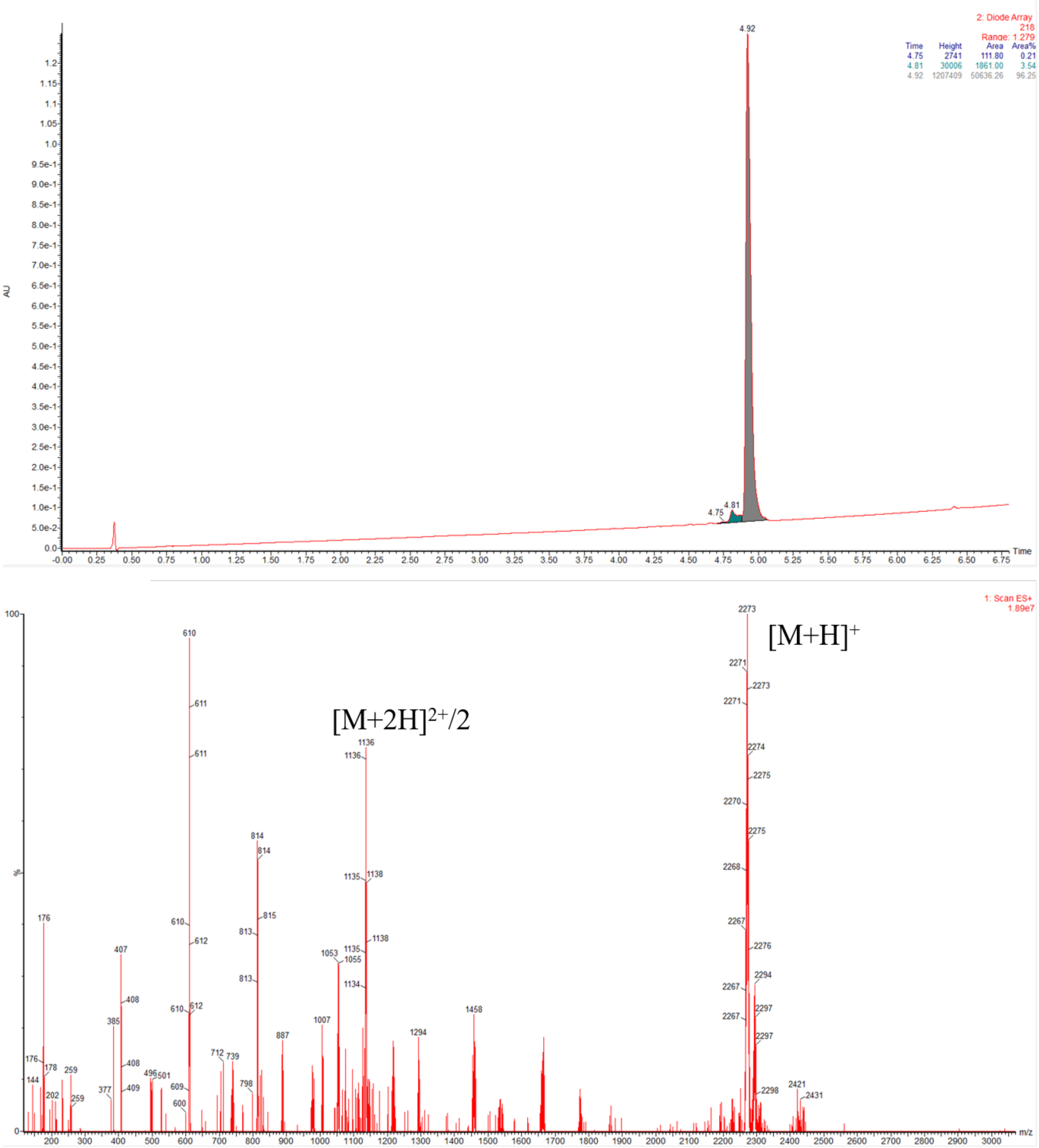

Fig. S10. UPLC-MS trace of purified Nte$_4$-N3Brpe$_6$ (**10**) with gradient of 20-80% ACN in H_2O in 6.8 min. LC trace of **10** (top image) and mass spectrum of **10** (bottom image).

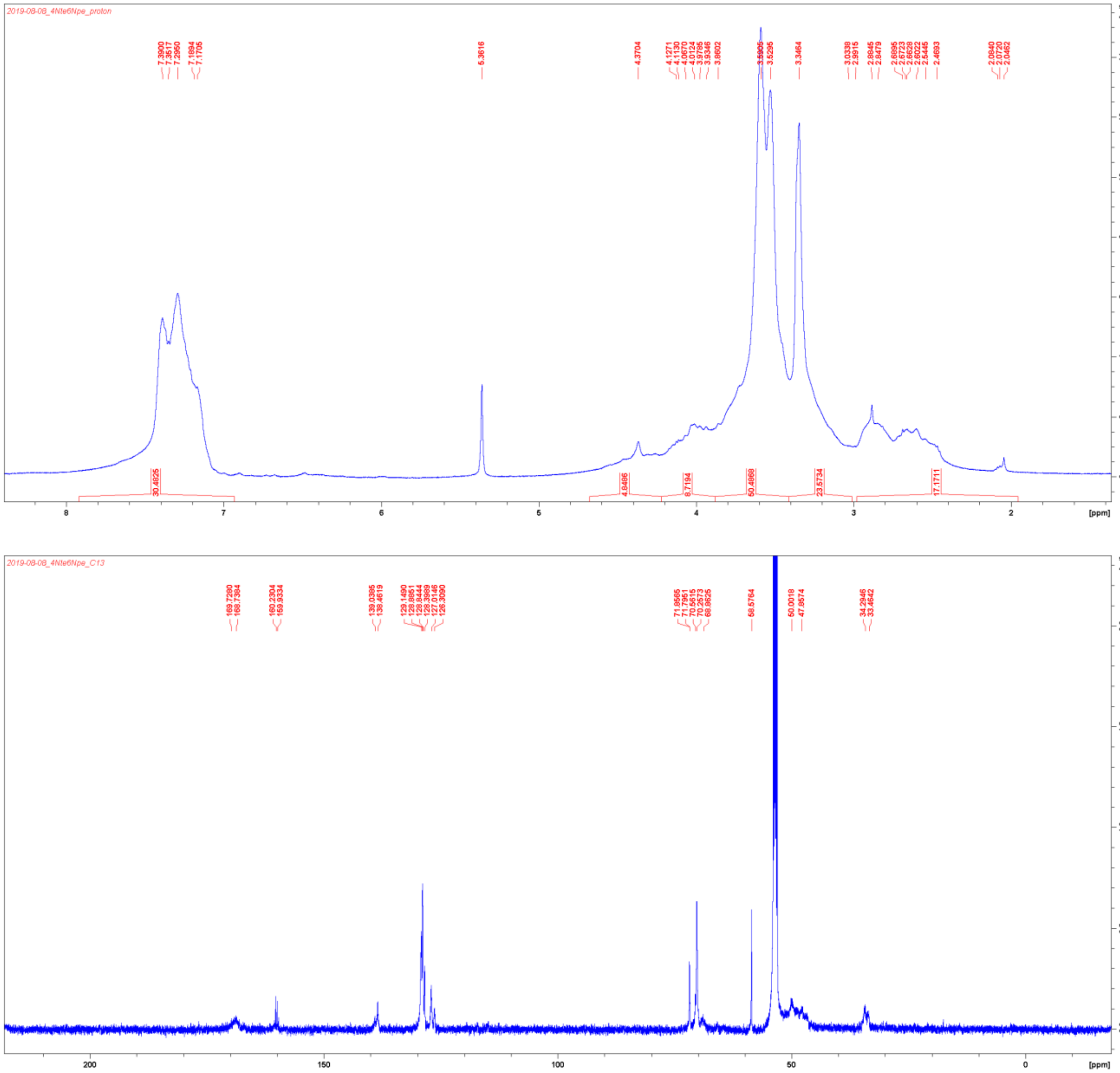

Fig. S11. ^{1}H and ^{13}C NMR spectra of Nte$_4$-N4pe$_6$ (**1**) in CD_2Cl_2.

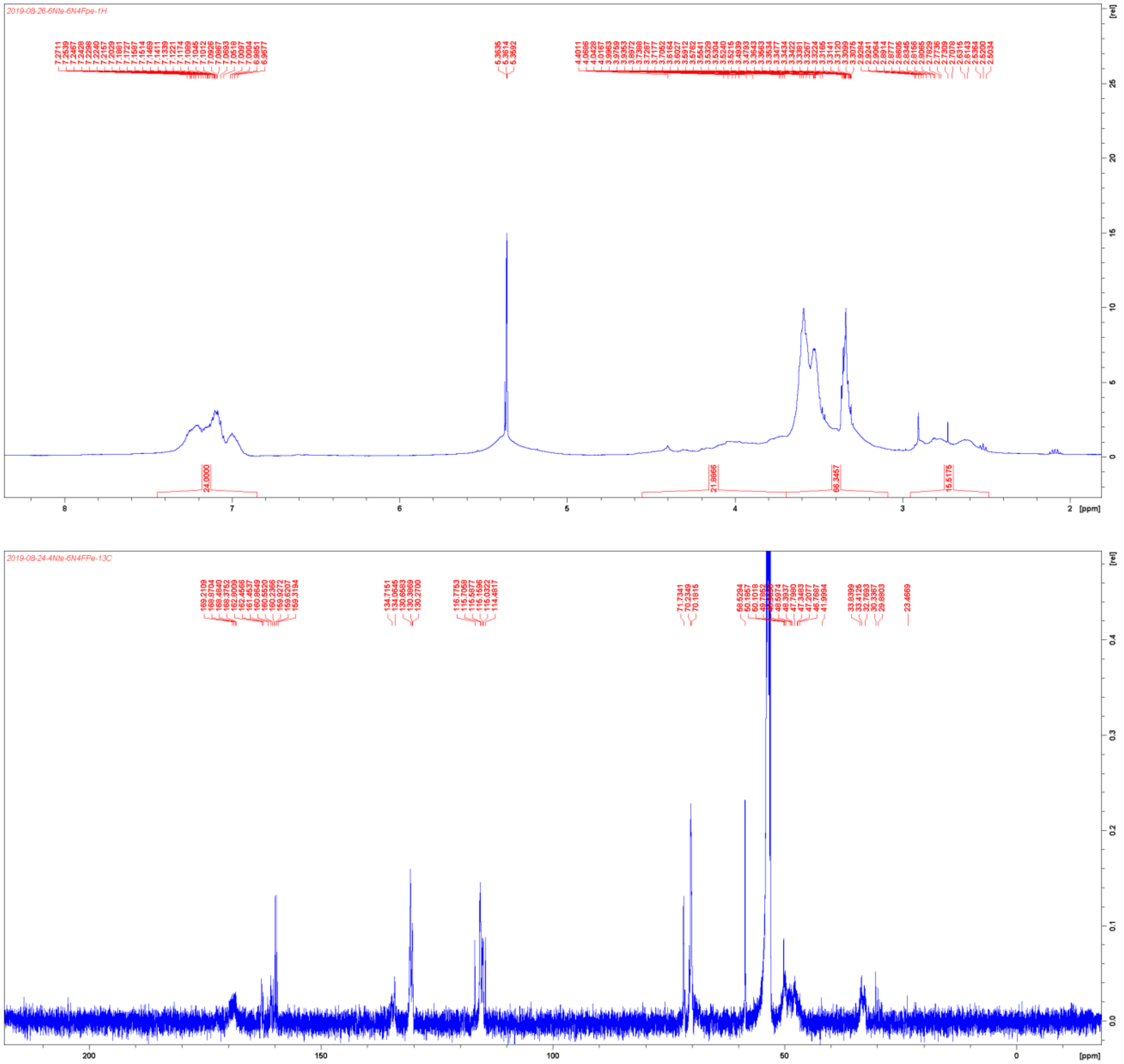

Fig. S12. ^{1}H and ^{13}C NMR spectra of Nte_4-$N4Fpe_6$ (**2**) in CD_2Cl_2.

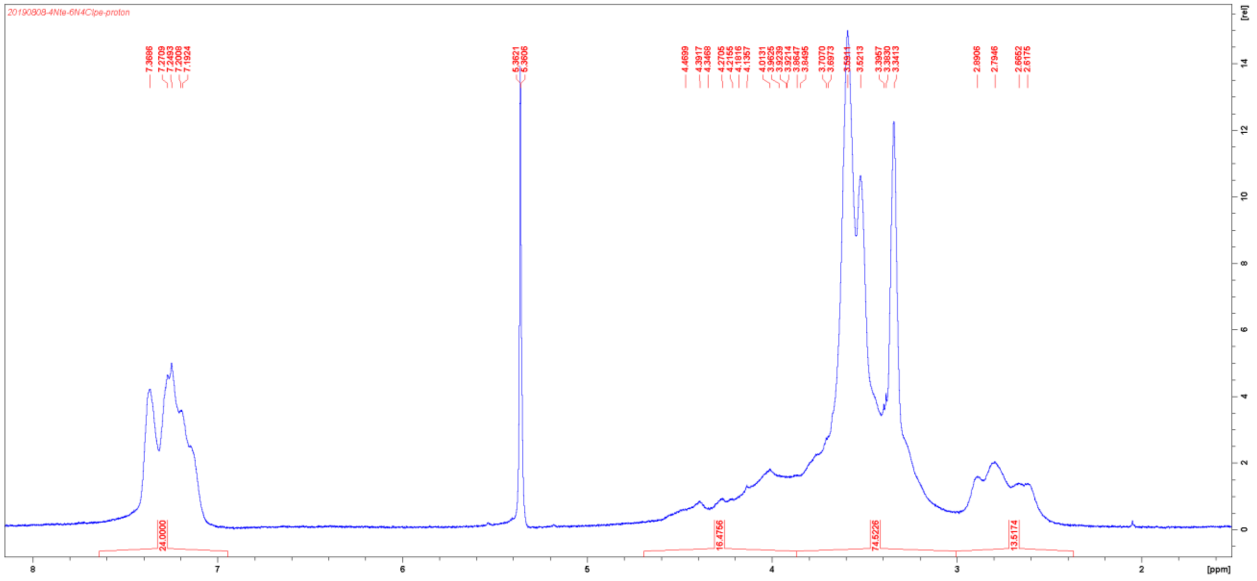

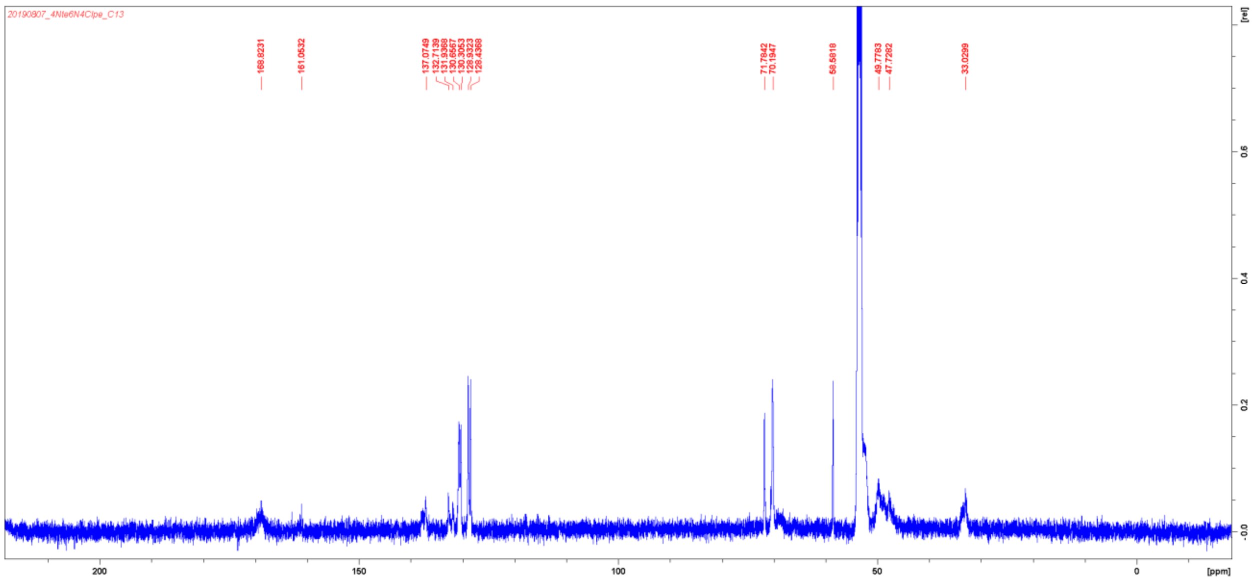

Fig. S13. ^{1}H and ^{13}C NMR spectra of Nte_4-N4$Clpe_6$ (**3**) in CD_2Cl_2.

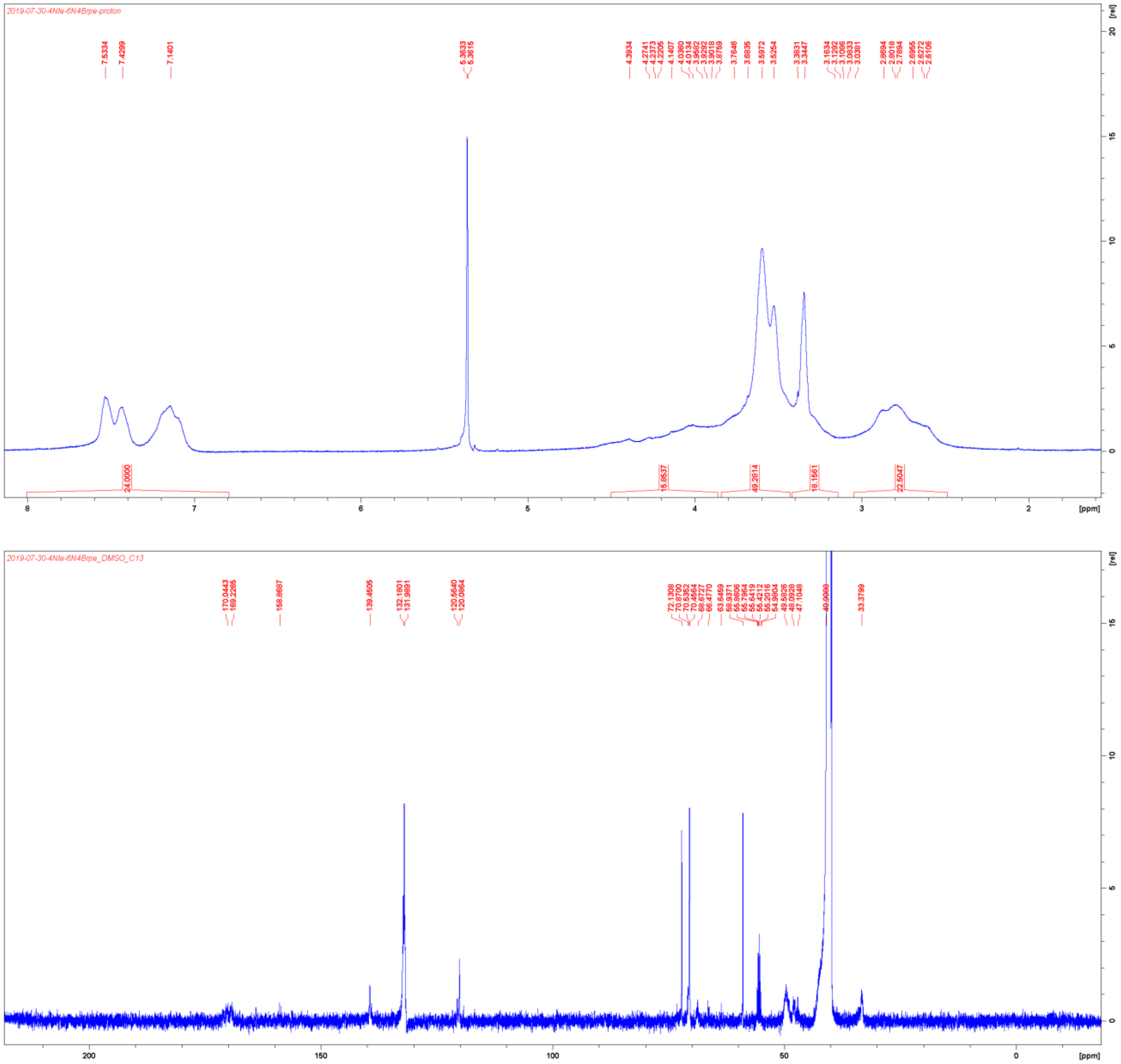

Fig. S14. ^{1}H and ^{13}C NMR spectra of Nte$_4$-N4Brpe$_6$ (**4**) in CD_2Cl_2.

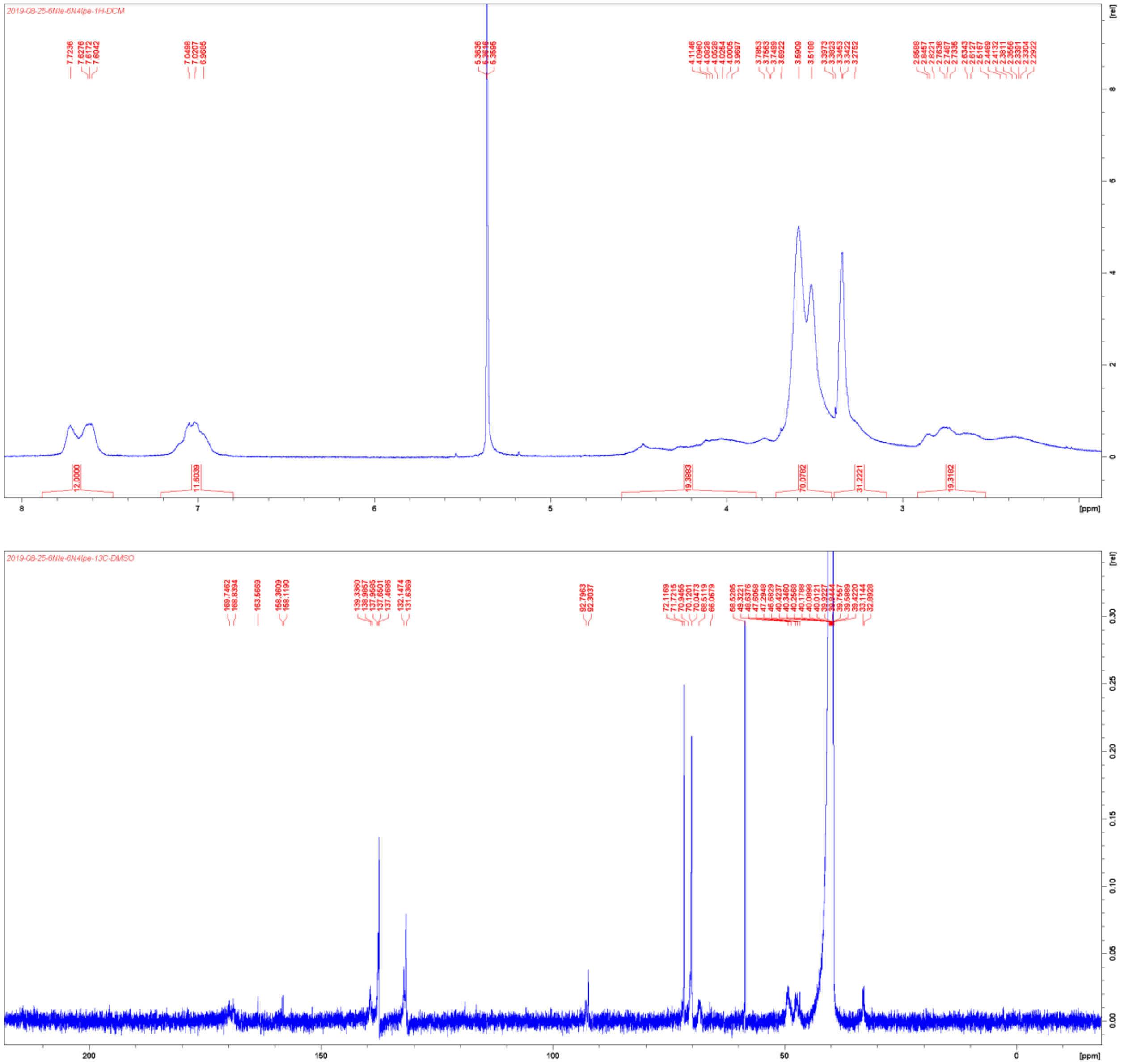

Fig. S15. ^{1}H and ^{13}C NMR spectra of Nte$_6$-N4Ipe$_6$ (**5**) in CD_2Cl_2 and DMSO-d^6, respectively. The ^{13}C NMR was conducted in DMSO-d^6 due to limited solubility at high concentration in CD_2Cl_2.

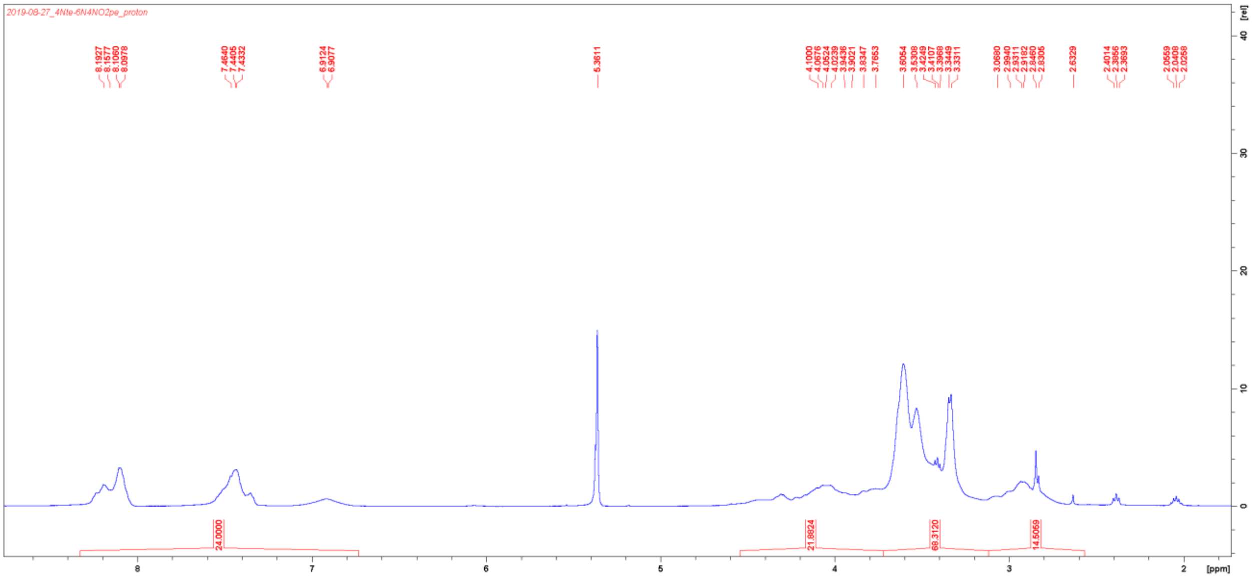

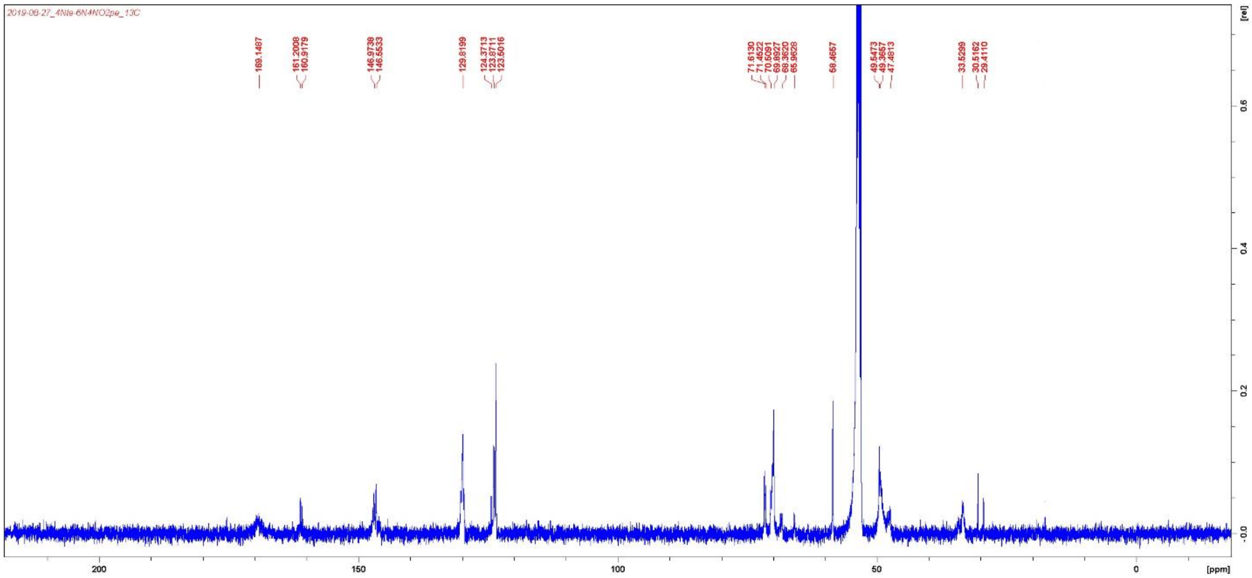

Fig. S16. ^{1}H and ^{13}C NMR spectra of Nte_4-N4NO2pe_6 (**6**) in CD_2Cl_2.

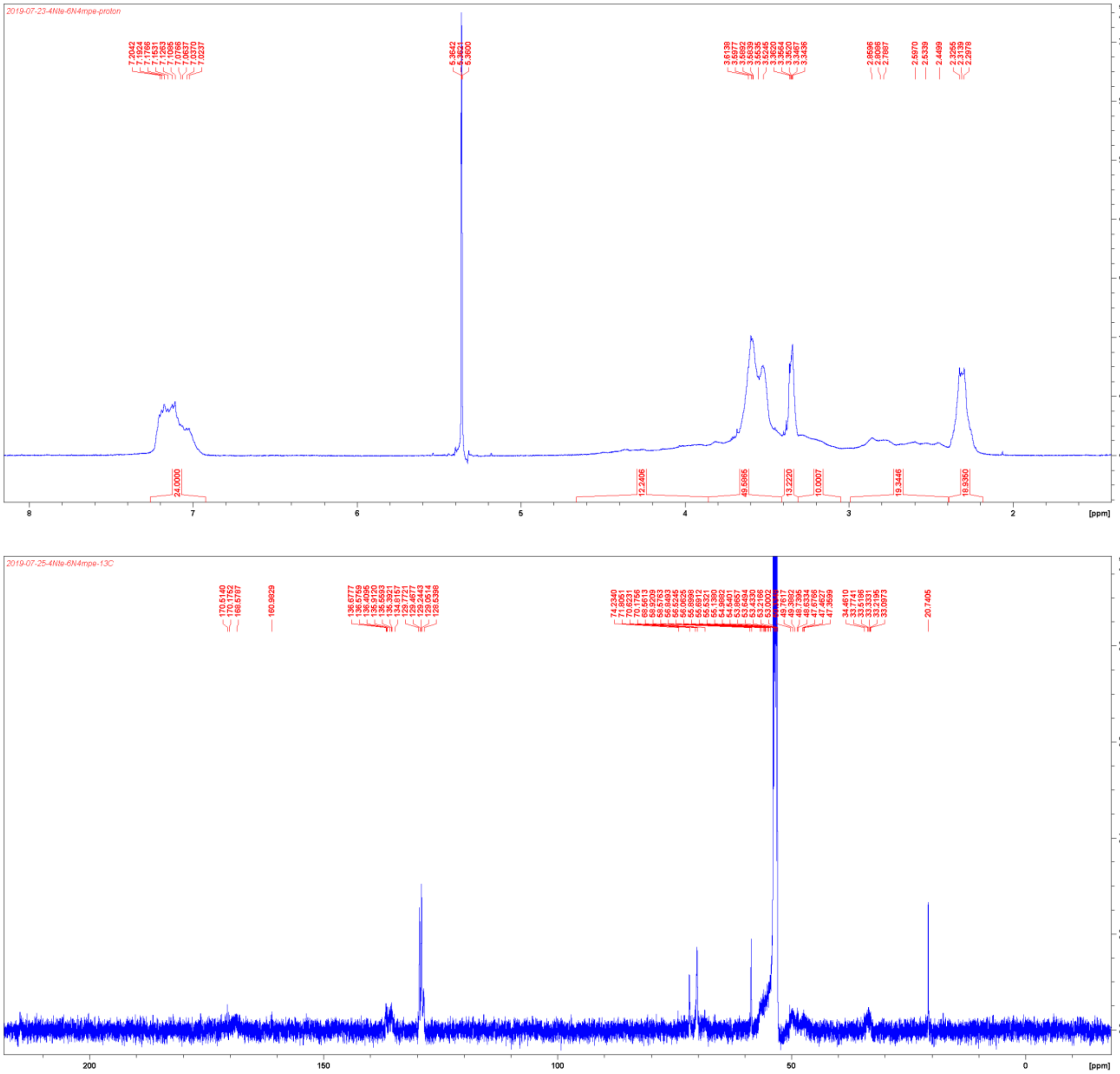

Fig. S17. ^{1}H and ^{13}C NMR spectra of Nte$_4$-N4mpe$_6$ (**7**) in CD_2Cl_2.

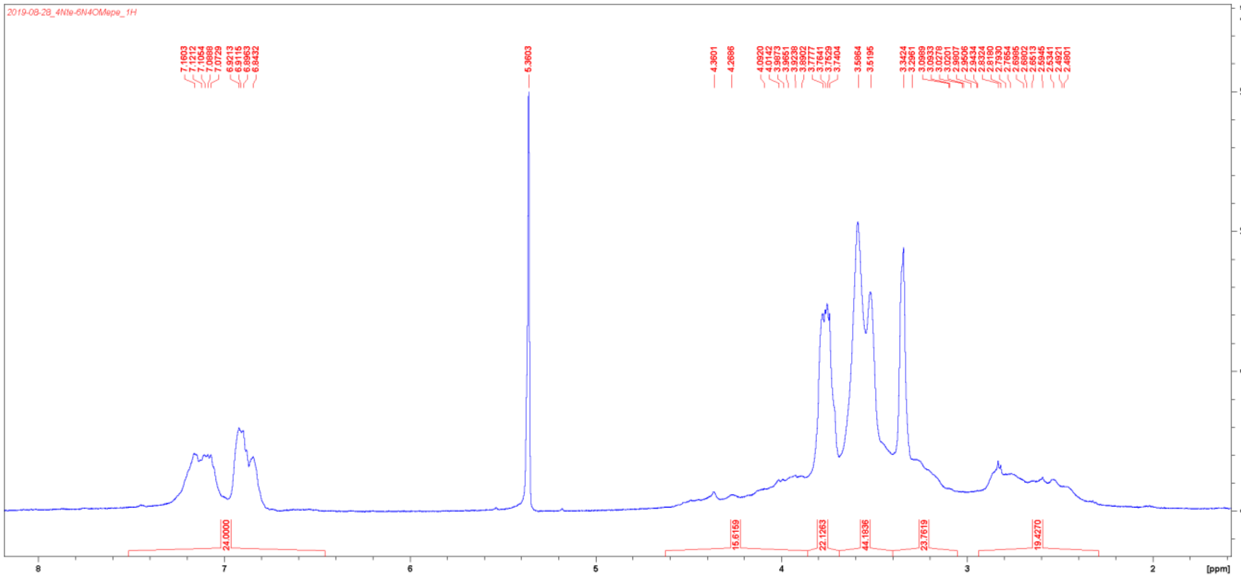

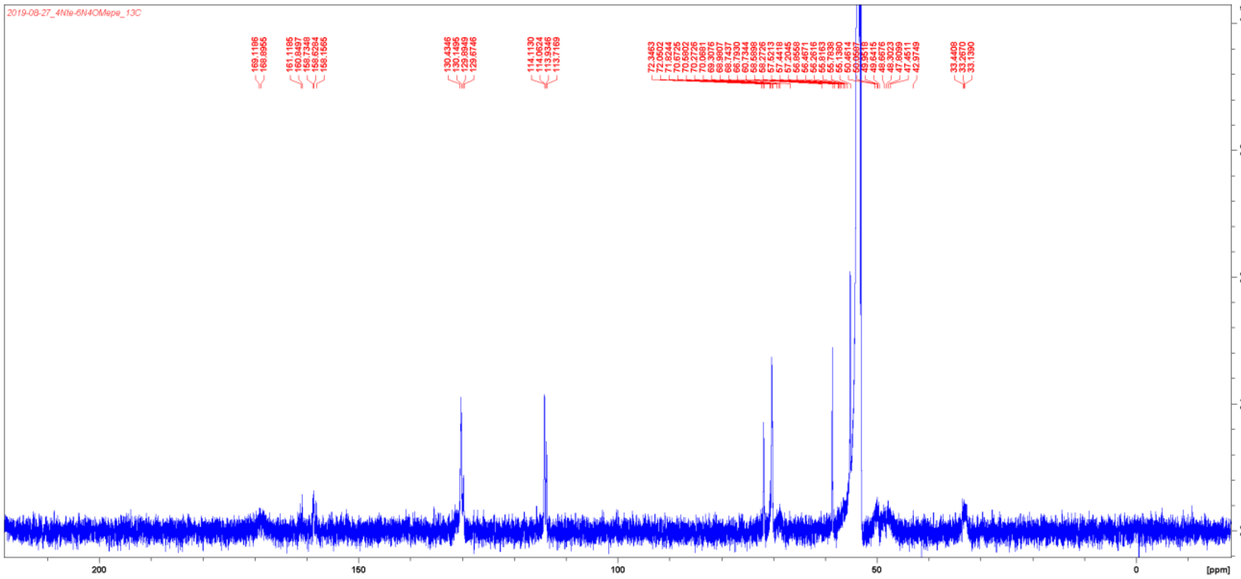

Fig. S18. ^{1}H and ^{13}C NMR spectra of Nte_4-N4OMepe$_6$ (**8**) in CD_2Cl_2.

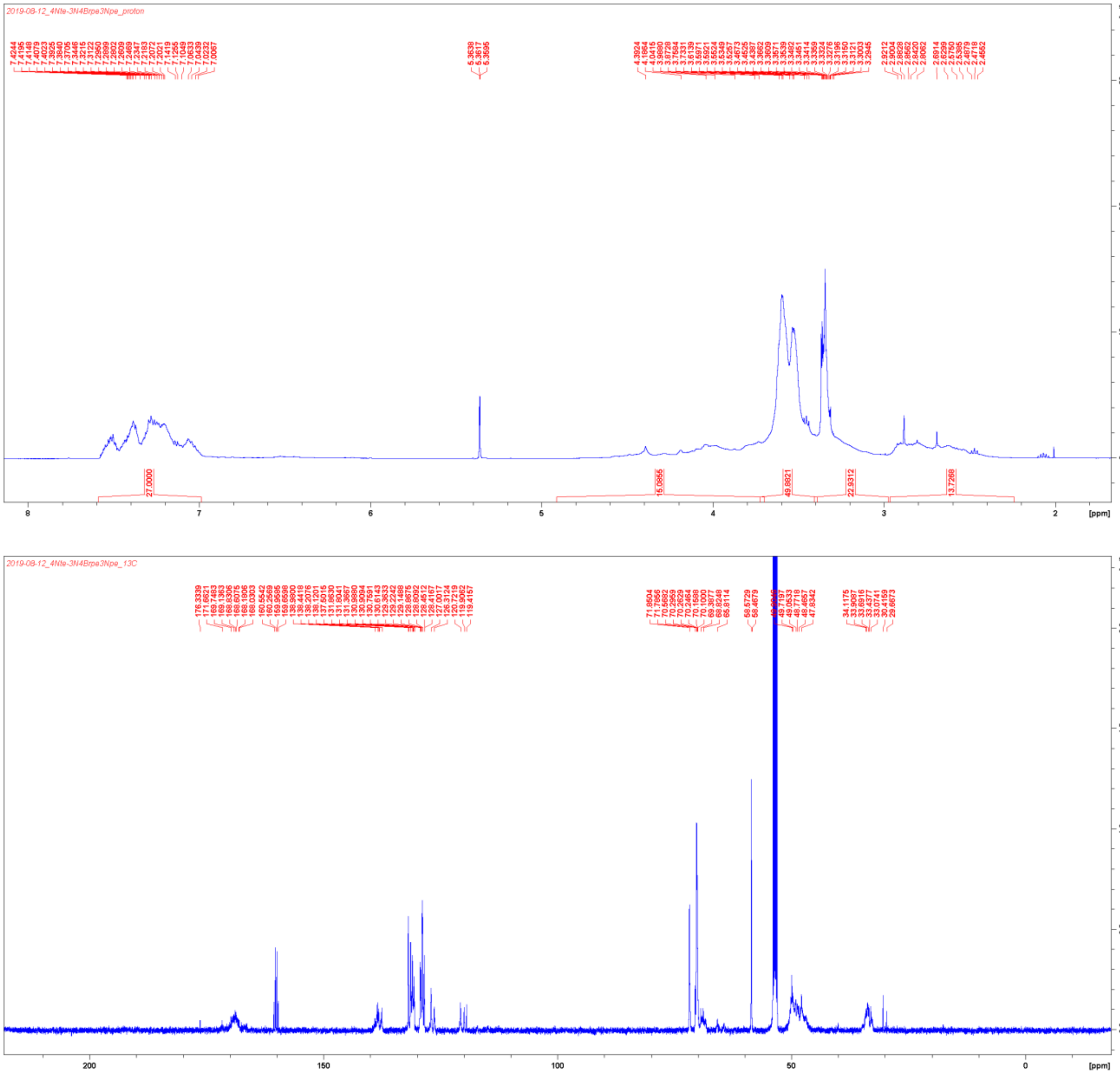

Fig. S19. ^{1}H and ^{13}C NMR spectra of Nte_4-($N4Brpe_6Npe)_3$ (**9**) in CD_2Cl_2.

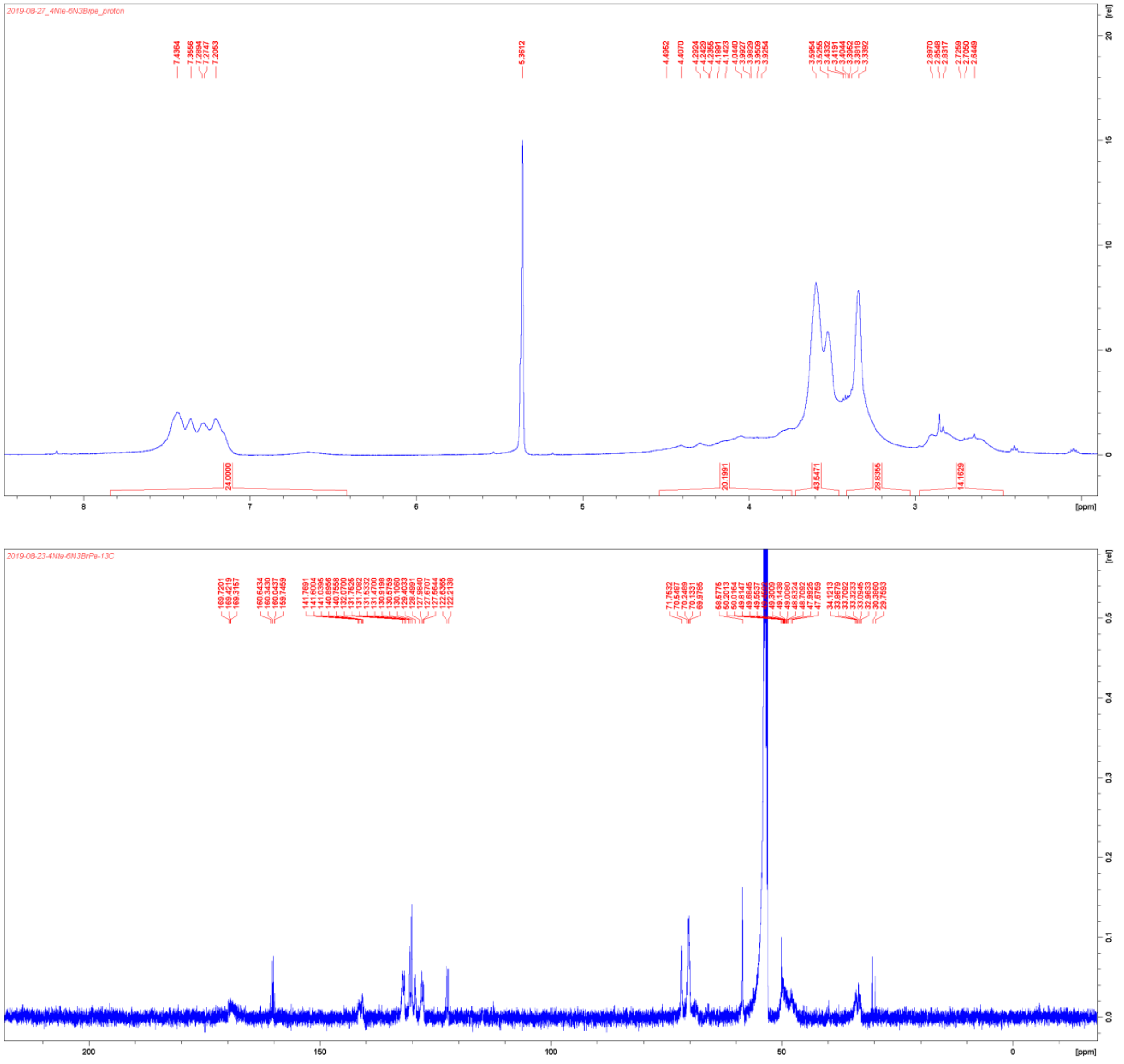

Fig. S20. ^{1}H and ^{13}C NMR spectra of Nte$_4$-N3Brpe$_6$ (**10**) in CD_2Cl_2.

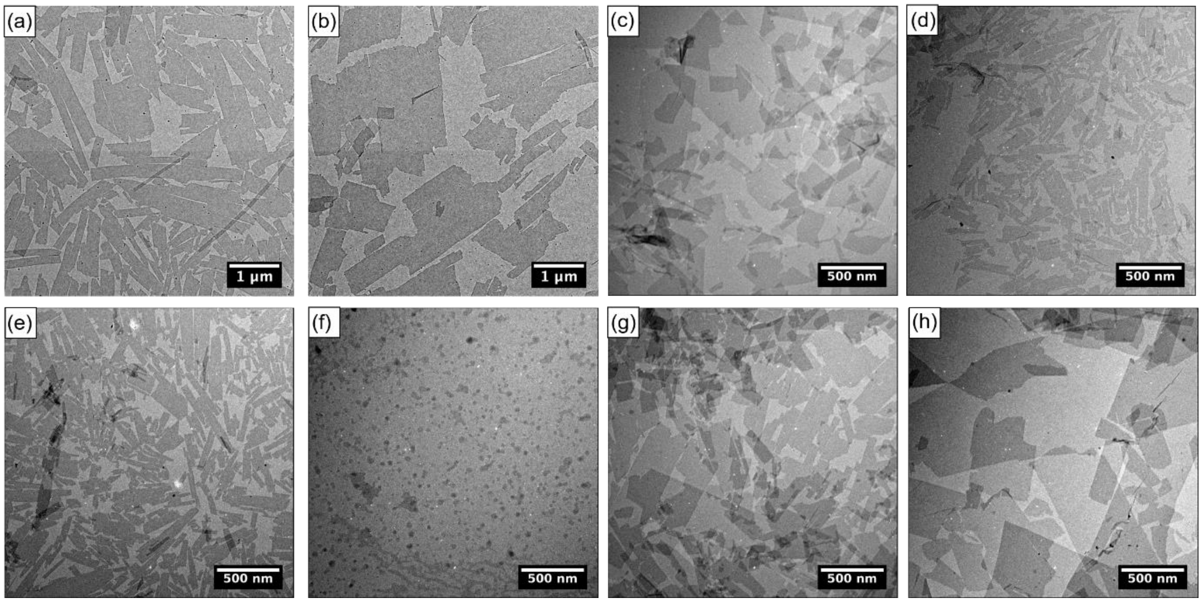

Fig. S21. TEM images of dry sheet Nte$_4$-N4Brpe$_6$ (**4**) (a), Nte$_4$-(N4BrpeNpe)$_3$ (**9**) (b), Nte$_4$-N4Fpe$_6$ (**2**) (c), Nte$_4$-N4Clpe$_6$ (**3**) (d), Nte$_6$-N4Ipe$_6$ (**5**) (e), Nte$_4$-N4NO$_2$pe$_6$ (**6**) (f), and Nte$_4$-N4OMepe$_6$ (**8**) (g) and Nte$_6$-N3Brpe$_6$ (**10**).

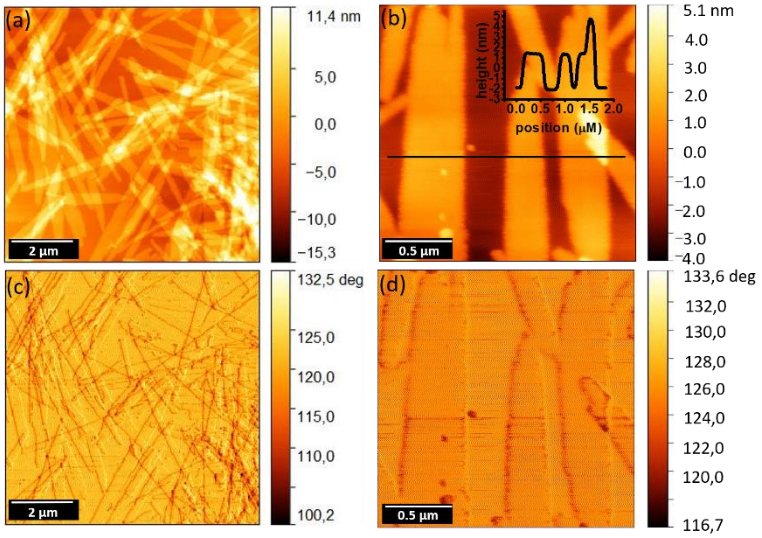

Fig. S22. AFM images of dry sheet Nte$_4$-Npe$_6$ (**1**): (a, b) height image; (c, d) corresponding phase image. The inset graph in (b) is the thickness profile of the sheets.

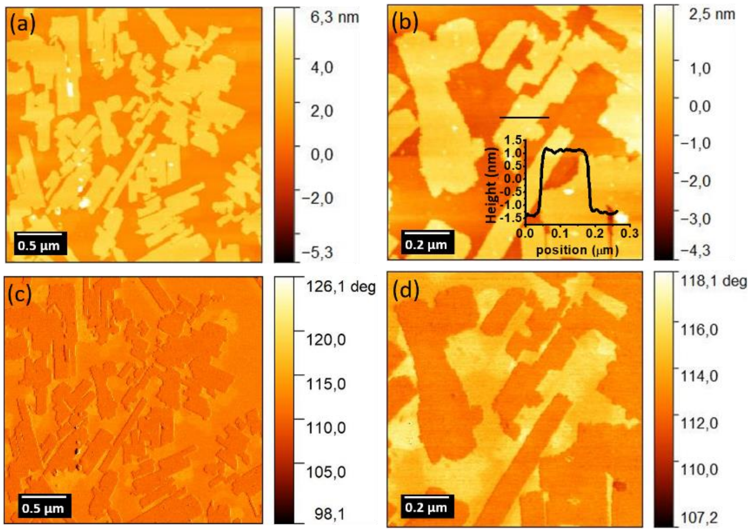

Fig. S23. AFM images of dry sheet Nte$_4$-N4Brpe$_6$ (**4**): (a, b) height images; (c, d) corresponding phase images. The inset graph in (b) is the thickness profile of the sheet.

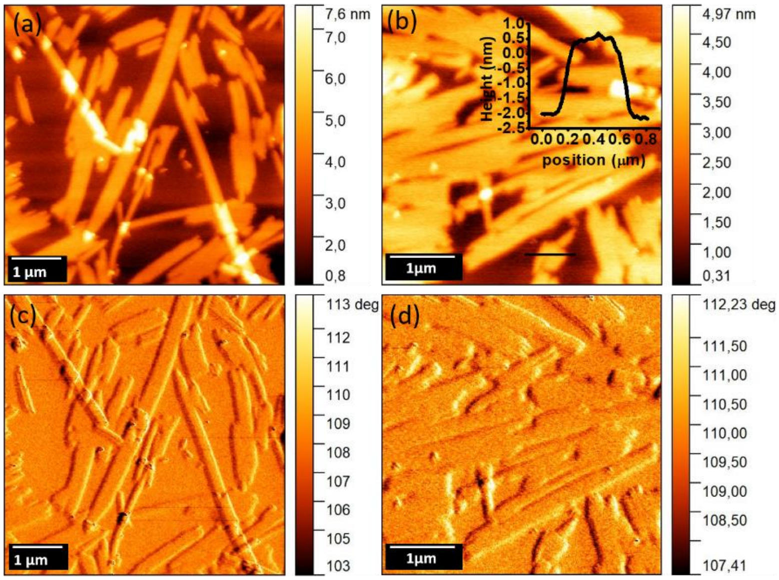

Fig. S24. AFM images of dry sheet Nte$_4$-N4mpe$_6$ (**7**): (a, b) height images; (c, d) corresponding phase images. The inset graph in (b) is the thickness profile of the sheet.

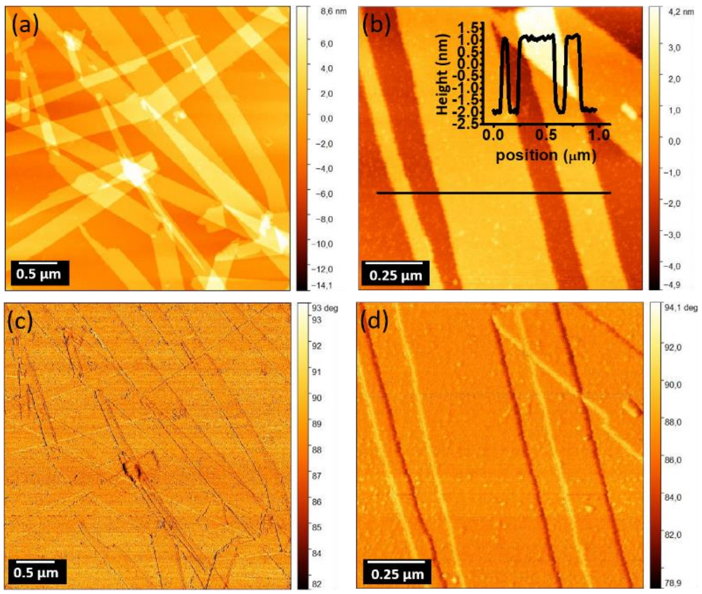

Fig. S25. AFM images of dry sheet Nte_4-$(N4BrpeNpe)_3$ (**9**): (a, b) height images; (c, d) corresponding phase images. The inset graph in (b) is the thickness profile of the sheets.

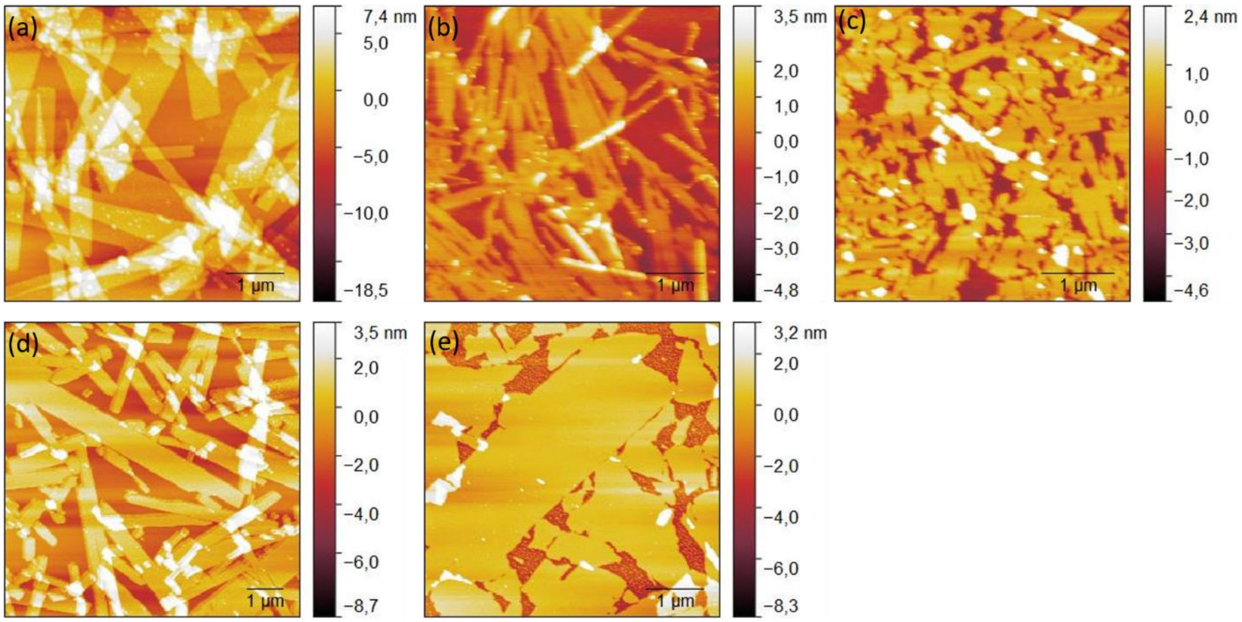

Fig. S26. AFM images of dry sheet Nte_4-$N4Fpe_6$ (**2**) (a), Nte_4-$N4Clpe_6$ (**3**) (b), Nte_6-$N4Ipe_6$ (**5**) (c), Nte_4-$N4OMepe_6$ (**8**) (d), and Nte_4-$N3Brpe_6$ (**10**) (e).

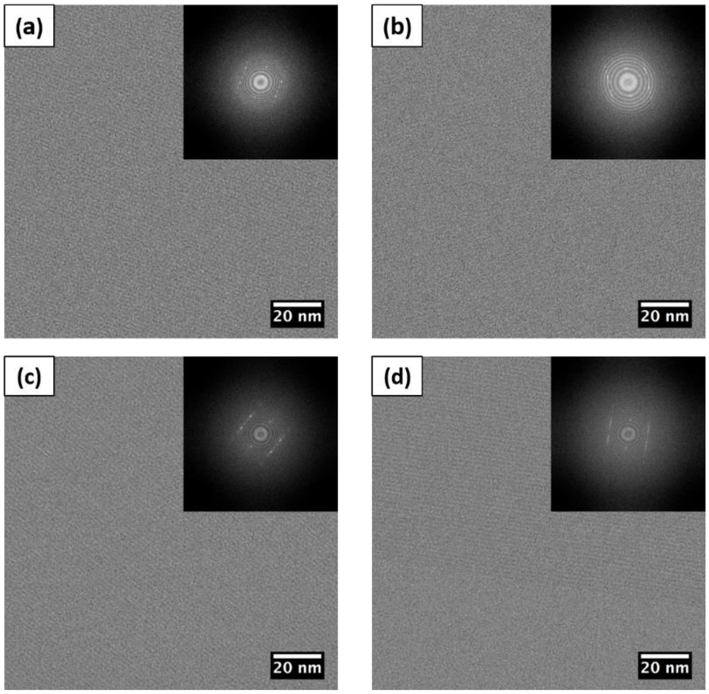

Fig. S27. Low-dose cryo-TEM micrographs of nanosheets. (a) Nte_4-Npe_6. (b) Nte_4-$N4Brpe_6$. (c) Nte_4-$N4mpe_6$. (d) Nte_4-$(N4BrpeNpe)_3$. Dark areas in all images represent the electron dense regions. Insets show the FFTs of the low-dose cryo-TEM micrographs with reflections and Thon rings due to the thin carbon support.

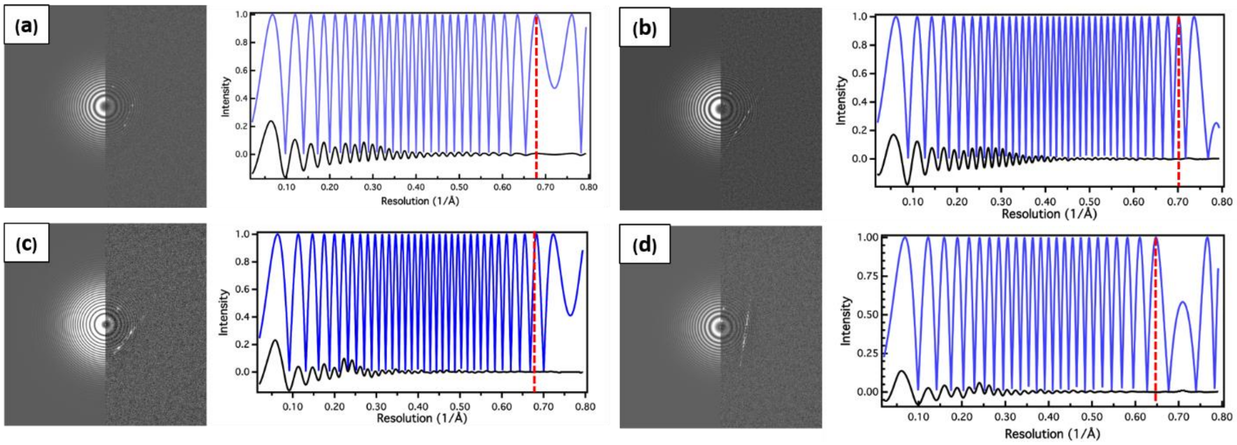

Fig. S28. CTF estimation of low-dose cryo-TEM micrographs**.** (a) Nte_4-Npe_6. (b) Nte_4-$N4Brpe_6$. (c) Nte_4-$N4mpe_6$. (d) Nte_4-$(N4BrpeNpe)_3$. Left panel shows the simulated CTF and the FFT of a selected micrograph. Right panel shows the 1-D simulated CTF plot (blue) and background subtracted Thon rings (black). Red dash line indicates the best resolution of CTF where the Thon ring can be fitted reasonably.

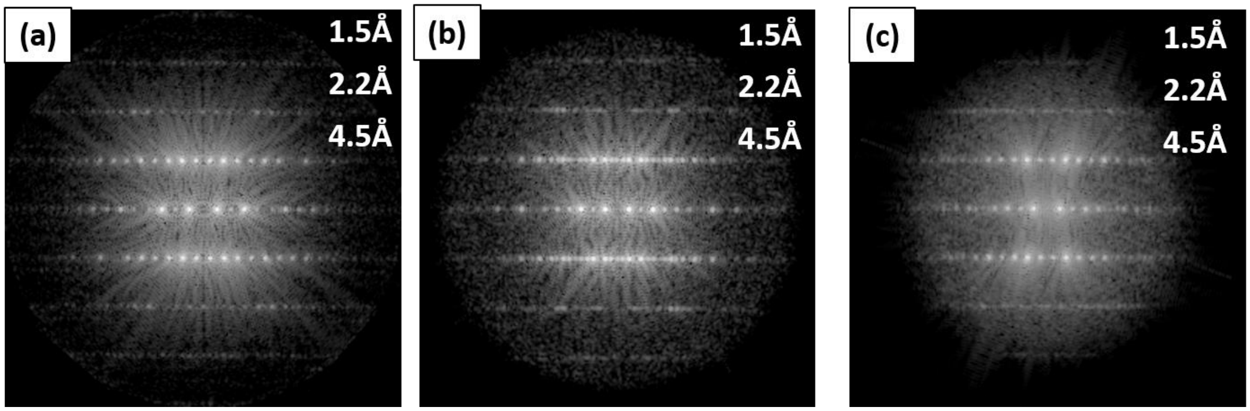

Fig. S29. FFTs of averaged images shown in Figure 2c, Figure 3c and Figure 4c: (a) Nte_4-Npe_6; (b) Nte_4-$N4Brpe_6$; (c) Nte_4-$(N4BrpeNpe)_3$.

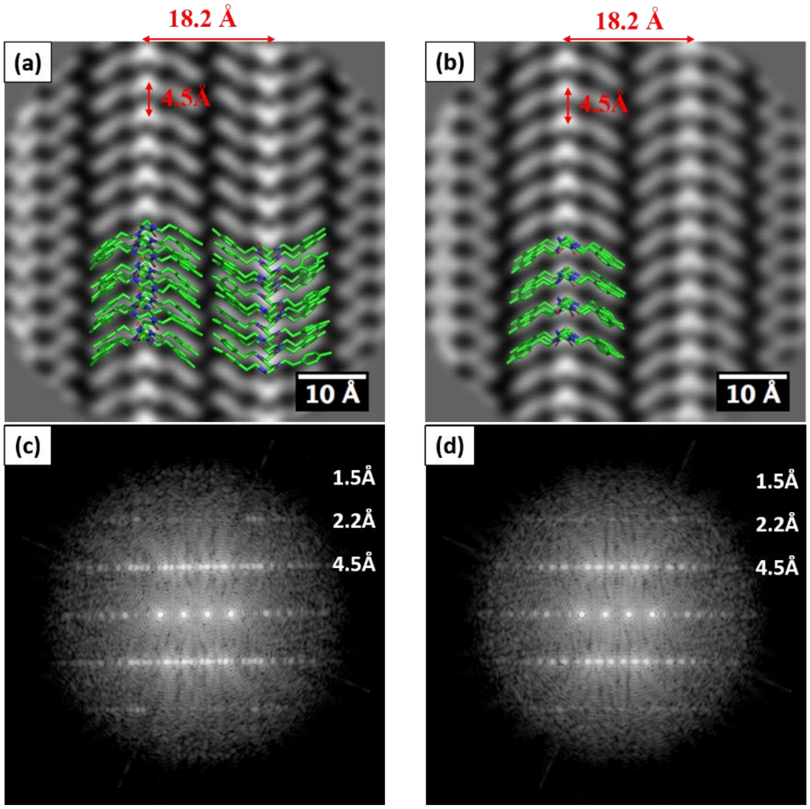

Fig. S30. Averaged images of sheet Nte_4-$N4mpe_6$ (**7**) showing both anti-parallel (a) and parallel (b) V shapes. (c, d) FFTs of averaged images shown in (a) and (b), respectively.

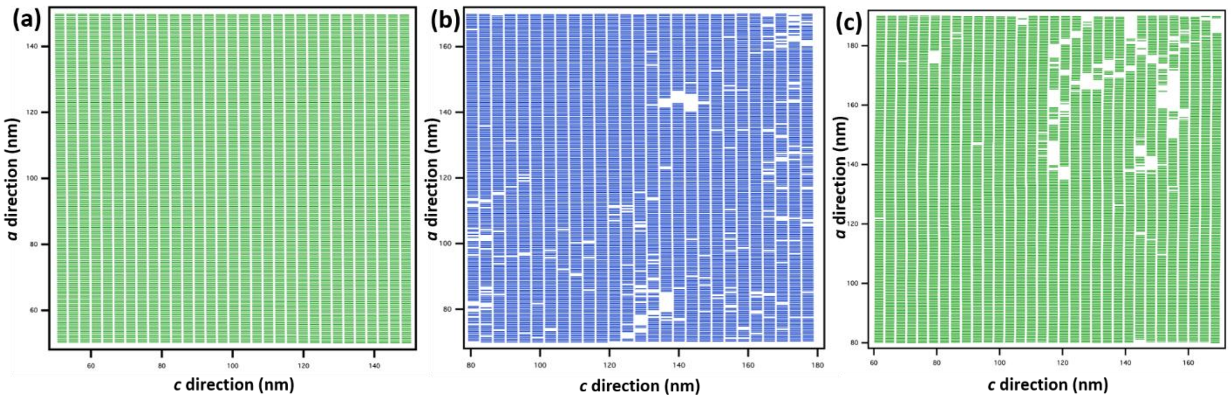

Fig. S31. Distribution maps of motifs in the nanosheets**.** Each individual rectangle in the maps represents a unit cell. Anti-parallel V-shaped motif are shown as blue boxes. Parallel V-shaped motif are shown as green boxes. White areas within the maps represent the regions that could not be analyzed. (a) nanosheet Nte_4-Npe_6 (**1**). (b) nanosheet Nte_4-$N4Brpe_6$ (**4**). (c) nanosheet Nte_4-$(N4BrpeNpe)_3$ nanosheet (**9**).

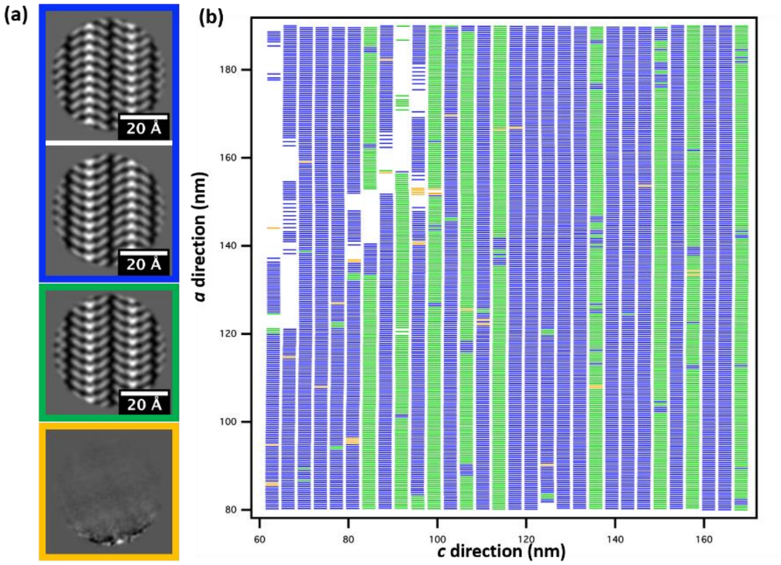

Fig. S32. Crystal motifs and distribution map in nanosheet Nte_4-$N4mpe_6$ (**7**). (a) Boxes were sorted and averaged assuming the presence of 4 classes. These classes fall naturally into two distinct groups that contain ordered motifs: anti-parallel V-shaped motifs (group 1 identified by a blue outline), and parallel V-shaped motifs (group 2 identified by a green outline). Class that includes is referred to as disordered (group 3 identified by a yellow outline). The *a* and *c* directions are specified for ordered motifs. Bright areas in all images represent the electron dense regions. (b)

Each individual rectangle in the maps represents a unit cell. Anti-parallel V-shaped motifs are shown as blue boxes. Parallel V-shaped motifs are shown as green boxes. Disordered motifs are shown as yellow boxes. White areas within the maps represent the regions that could not be analyzed.

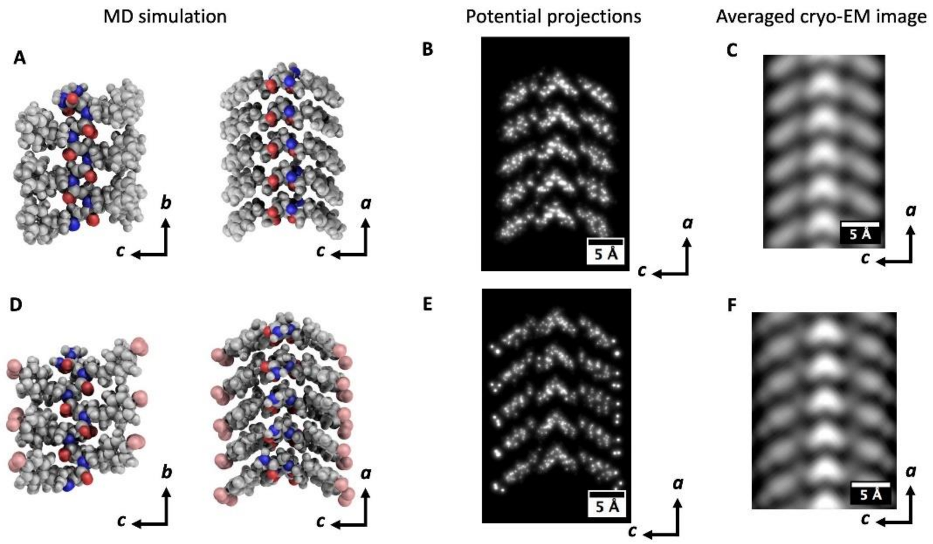

Fig. S33. Comparisons between simulations and cryo-TEM images. (a) MD simulations of Nte_4-Npe_6, only the crystallized Npe_6 block are shown. (b) Potential projection of the atomic model in A through *a*-*c* plane, it shows the electron density map of crystallized Npe_6 block. (c) A section of averaged cryo-TEM image shows identical features, including chevron shaped backbone, dihedral angle of benzene rings and similar contrast. (d) MD simulations of Nte_4-$N4Brpe_6$, only the crystallized $N4Brpe_6$ block are shown. (e) Potential projection of the atomic model in A through a-c plane, it shows the electron density map of crystallized $N4Brpe_6$ block. (f) A section of averaged cryo-TEM image shows identical features, including chevron shaped backbone, dihedral angle of benzene rings and addition contrast from the superposed bromine atoms appended to benzene rings. Nitrogen atoms and oxygen atoms are in blue and red in glycine backbones, respectively. Bromine atoms are in pink. Bright areas represent the electron dense regions in both projection maps and averaged images.

Table S2. Summary of properties of micrographs used in the analysis

Nanosheets	Number of micrographs	Defocus values applied in micrographs [a]	Number of boxes extracted from the micrographs	Highest resolution of Thon rings in the FFTs of micrographs [a]	Highest resolution of reflections in the FFTs of averaged images
Nte_4-Npe_6	4	-4830 Å to -5412 Å	102282	1.4 Å	1.5 Å
Nte_4-$N4Brpe_6$	4	-2790 Å to -4903 Å	88667	1.5 Å	1.5 Å
Nte_4-$N4mpe_6$	4	-4024 Å to -5698 Å	40712	1.5 Å	2.1 Å
Nte_4-$(N4BrpeNpe)_3$	3	-4381 Å to -5126 Å	60928	1.5 Å	1.5 Å

[a.] Determined by gCTF

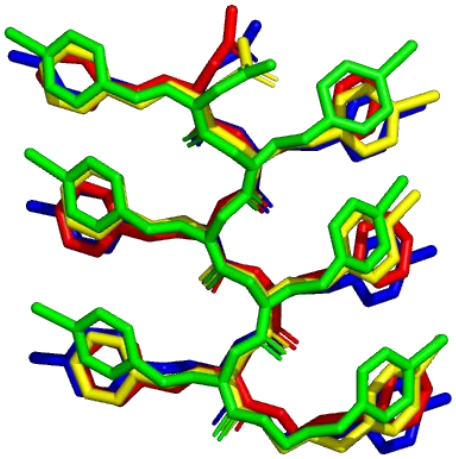

Fig. S34. Overlapped peptoid chains taken from the MD sheet models showing the same backbone fold (only hydrophobic block was shown): Npe_6 (red), $N4Brpe_6$ (green), $N4mpe_6$ (blue) and $(N4BrpeNpe)_3$ (yellow).

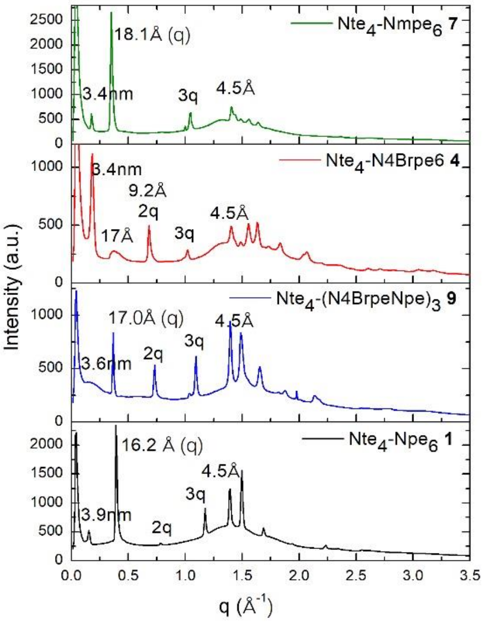

Fig. S35. XRD measurements of dry sheets **1**, **4**, **7** and **9**.

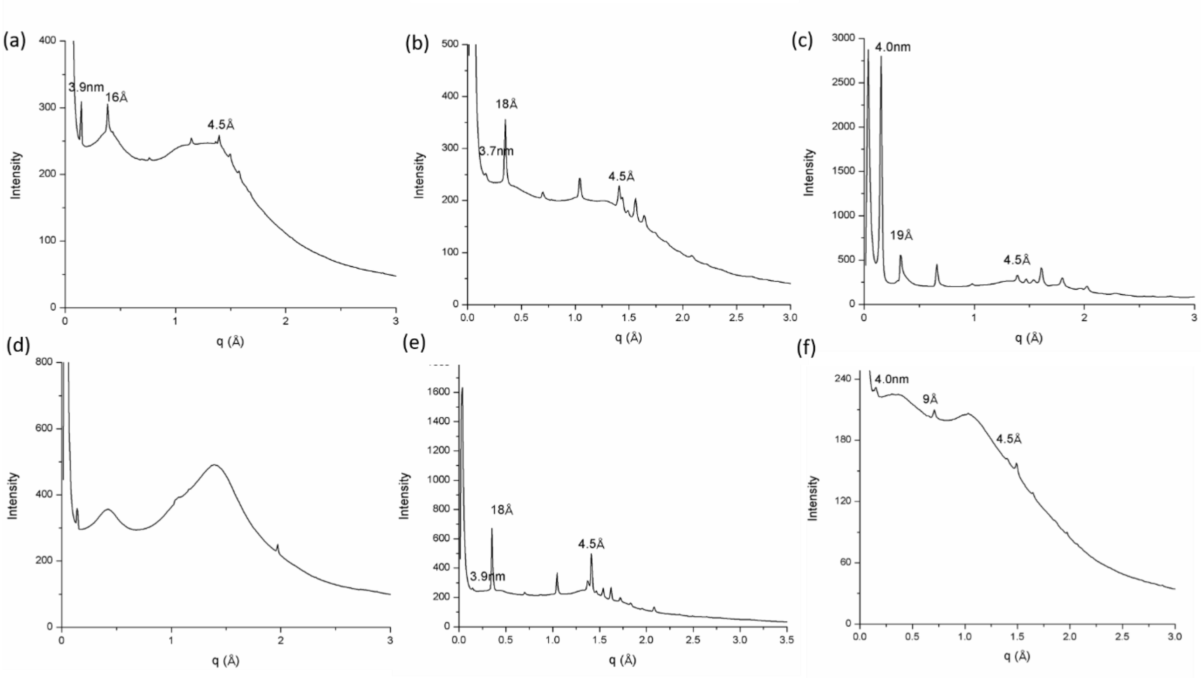

Fig. S36. XRD measurements of dry sheets **2** (a), **3** (b), **5** (c), **6** (d), **8** (e), **10** (f).

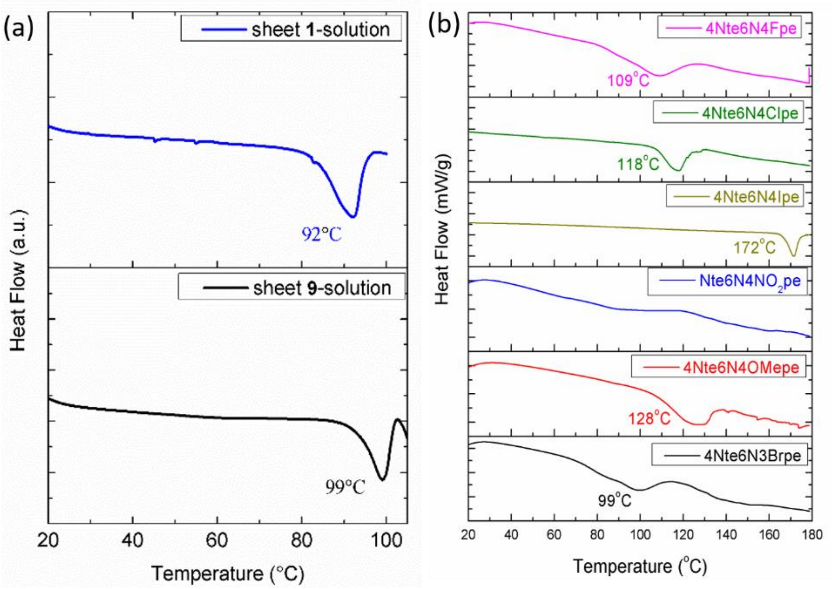

Fig. S37. NanoDSC measurements of sheets Nte4-Npe6 (**1**) and Nte4-(N4BrpeNpe)3 (**9**) in water at about 4mg/mL (a) and DSC measurements of dry sheets **2**, **3**, **5**, **6**, **8**, **10**.

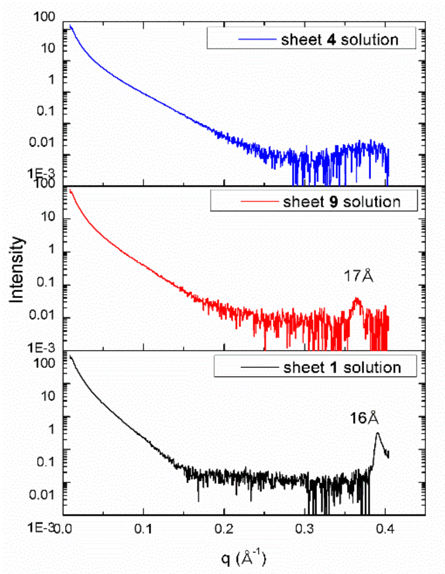

Fig. S38. SAXS measurements of sheets **1**, **4** and **9** in aqueous solutions.

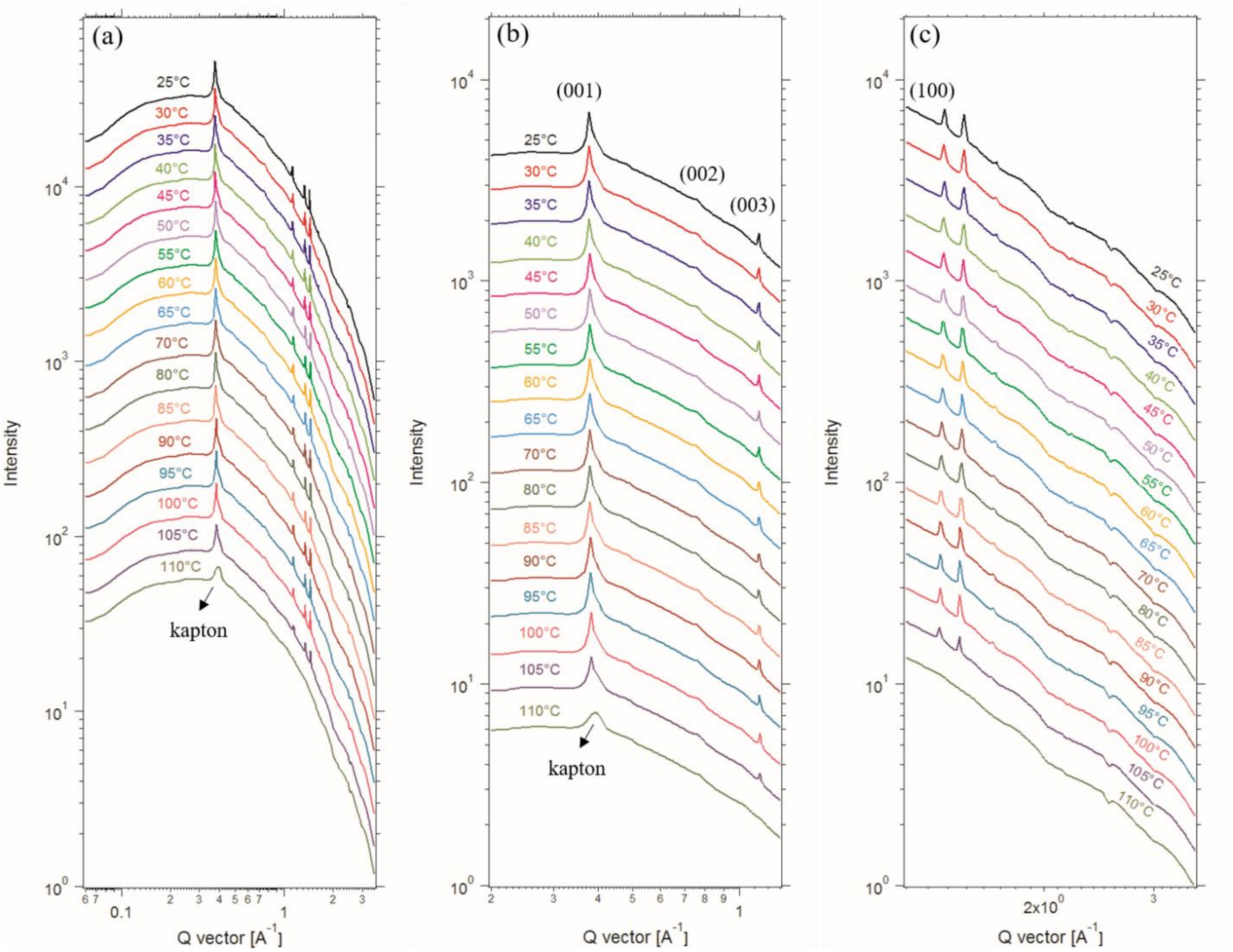

Fig. S39. WAXS measurements of sheet Nte_4-Npe_6 (**1**) as a function of increasing temperature**.** (a) WAXS measurements showing all the scattering peaks. (b) WAXS measurements showing the (001), (002) and (003) peaks corresponding to the *c* dimension. The peak at q = 0.4 Å^{-1} is from the Kapton windows. (c) WAXS measurements showing the peaks corresponding to the *a* dimension. At 110 ^{o}C, all the scattering peaks disappear simultaneously.

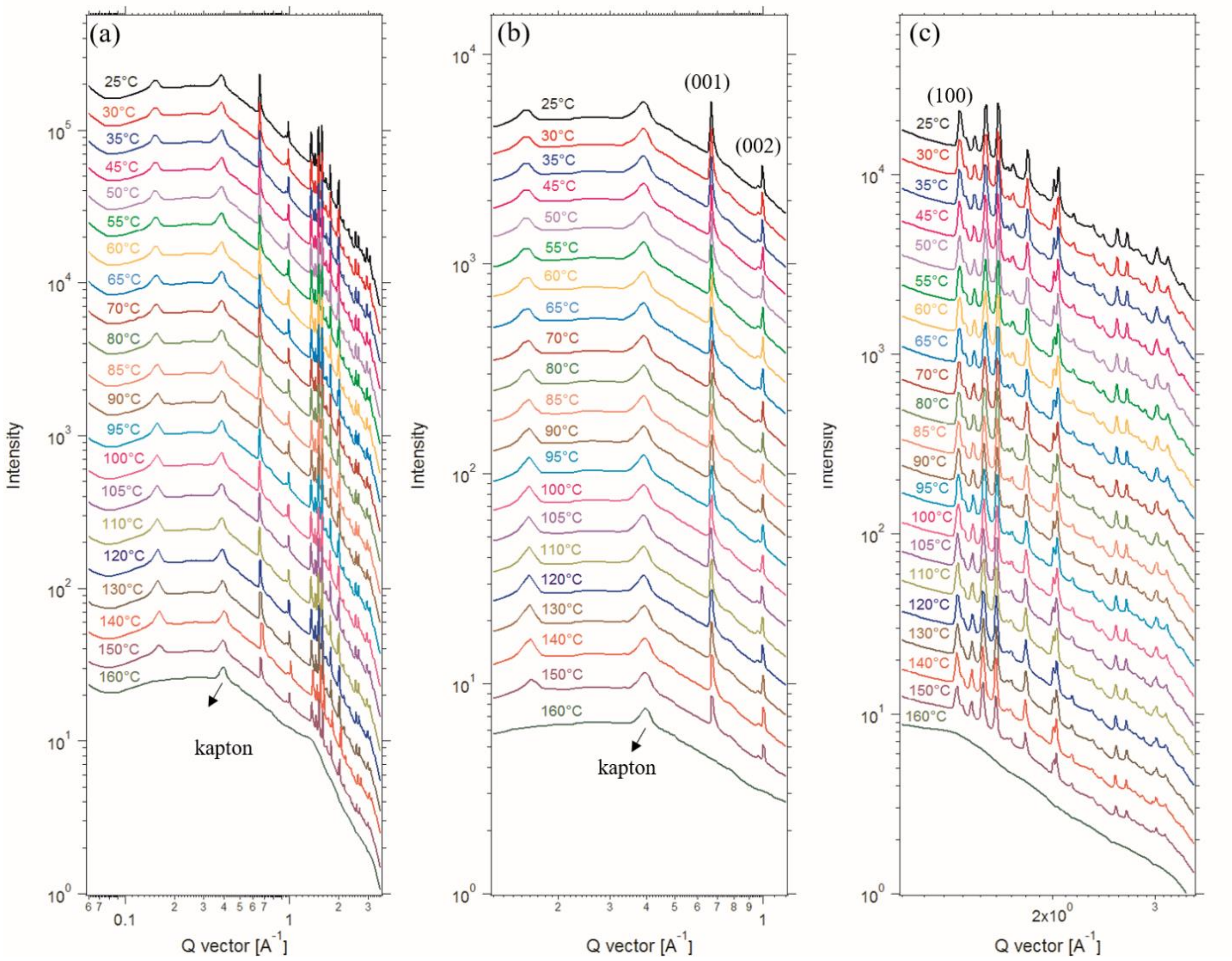

Fig. S40. WAXS measurements of sheet Nte$_4$-N4Brpe$_6$ (**4**) as a function of increasing temperature. (a) WAXS measurements showing all the scattering peaks. (b) WAXS measurements showing the (002) and (003) peaks corresponding to the *c* dimension. The peak at $q = 0.4$ Å^{-1} is from the Kapton windows. (c) WAXS measurements showing the peaks corresponding to the *a* dimension. At 160 ^{o}C, all the scattering peaks disappear simultaneously.

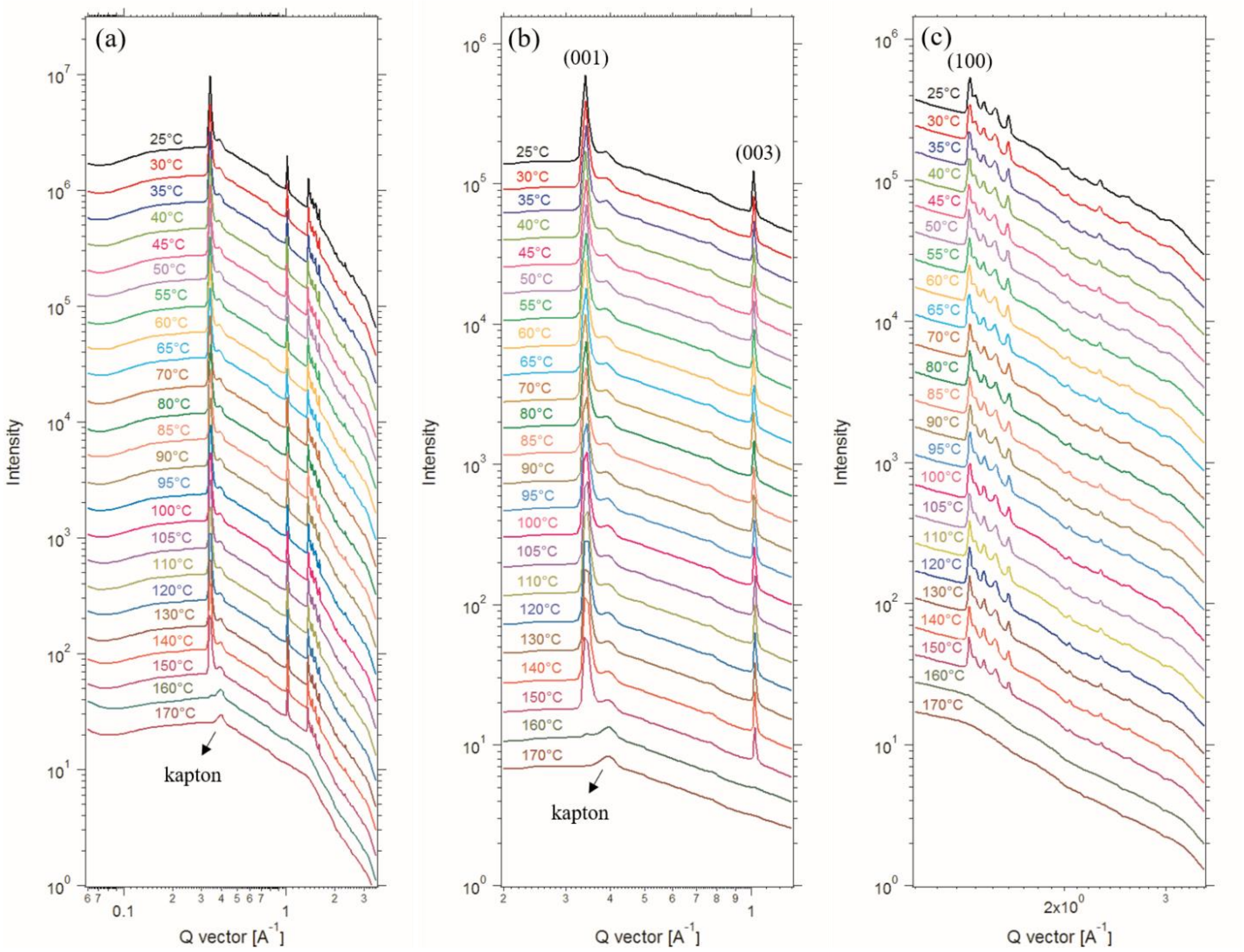

Fig. S41. WAXS measurements of dry sheet Nte_4-N4mpe_6 (**7**). as a function of increasing temperature. (a) WAXS measurements showing all the scattering peaks. (b) WAXS measurements showing the (001) and (003) peaks corresponding to the *c* dimension. The peak at q = 0.4 Å^{-1} is from the Kapton windows. (c) WAXS measurements showing peaks corresponding to the *a* dimension. At 170 ^{o}C, all the scattering peaks disappear simultaneously.

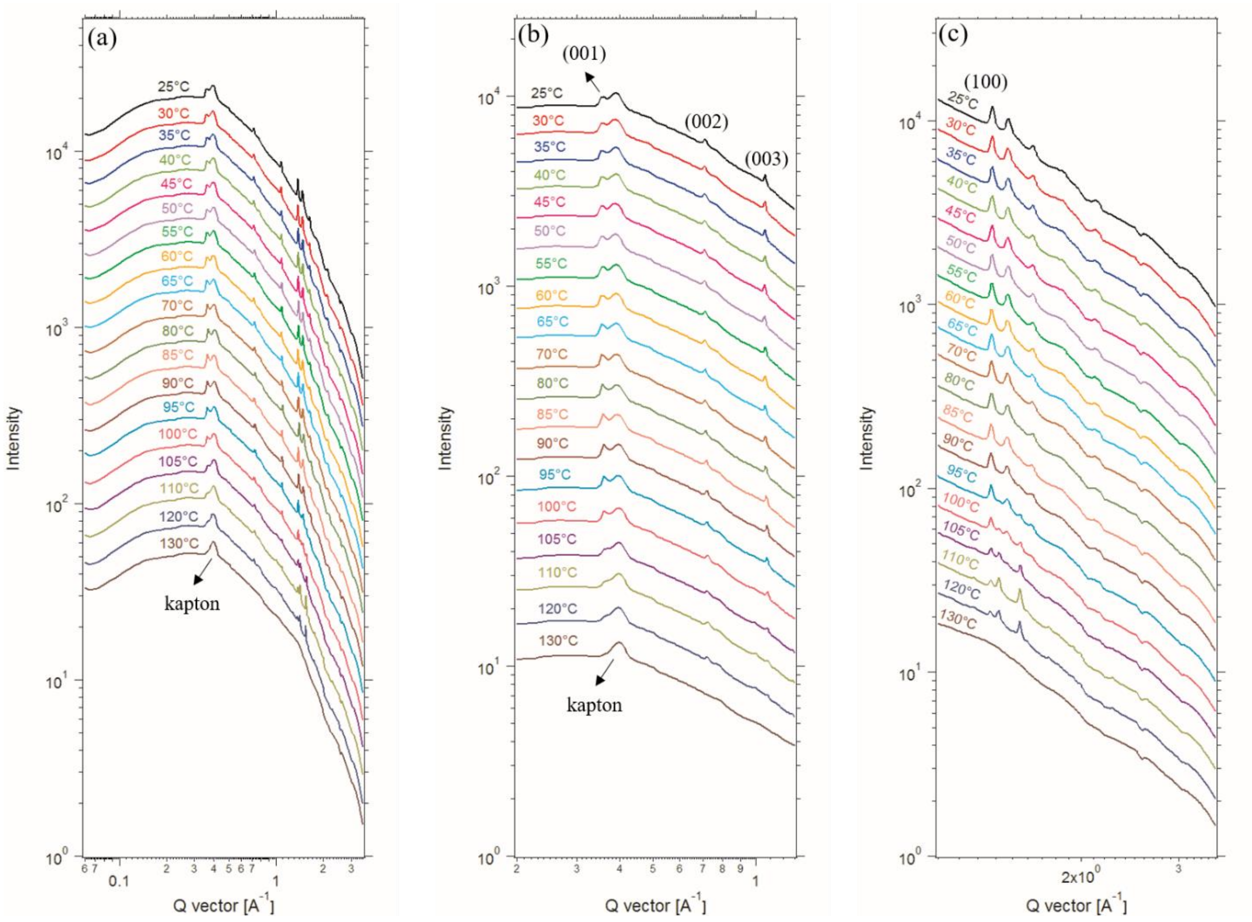

Fig. S42. WAXS measurements of dry sheet Nte$_4$-(N4BrpeNpe)$_3$ (**9**) as a function of increasing temperature. (a) WAXS measurements showing all the scattering peaks. (b) WAXS measurements showing the (001), (002) and (003) peaks corresponding to the *c* dimension. The peak at q = 0.4 Å^{-1} is from the Kapton windows. (c) WAXS measurements showing the peaks corresponding to the *a* dimension. At 130 ^{o}C, all the scattering peaks disappear simultaneously.

Reference

1. S. Q. Zheng, *et al.*, MotionCor2: anisotropic correction of beam-induced motion for improved cryo-electron microscopy. *Nat. Methods* **14**, 331 (2017).
2. K. Zhang, Gctf: Real-time CTF determination and correction. *J. Struct. Biol.* **193**, 1-12 (2016).
3. B. Gipson, X. Zeng, Z. Y. Zhang, H. Stahlberg, 2dx—User-friendly image processing for 2D crystals. *J. Struct. Biol.* **157**, 64-72 (2007).
4. B. Gipson, X. Zeng, H. Stahlberg, 2dx_merge: Data management and merging for 2D crystal images. *J. Struct. Biol.* **160**, 375-384 (2007).
5. M. Arheit, *et al.* "Image Processing of 2D Crystal Images" in Electron Crystallography of Soluble and Membrane Proteins: Methods and Protocols, I. Schmidt-Krey & Y. Cheng, Eds. (Humana Press, 2013), pp. 171-194.

6. R. Henderson, P. N. T. Unwin, Three-dimensional model of purple membrane obtained by electron microscopy. *Nature* **257**, 28-32 (1975).
7. R. Henderson, *et al.*, Structure of purple membrane from halobacterium halobium: recording, measurement and evaluation of electron micrographs at 3.5 Å resolution. *Ultramicroscopy* **19**, 147-178 (1986).
8. X. Jiang, *et al.*, Imaging Unstained Synthetic Polymer Crystals and Defects on Atomic Length Scales Using Cryogenic Electron Microscopy. *Macromolecules* **51**, 7794-7799 (2018).
9. S. H. W. Scheres, RELION: Implementation of a Bayesian approach to cryo-EM structure determination. *J. Struct. Biol.* **180**, 519-530 (2012).
10. S. H. W. Scheres, A Bayesian View on Cryo-EM Structure Determination. *J. Mol. Biol.* **415**, 406-418 (2012).
11. F. J. Sigworth, P. C. Doerschuk, J.-M. Carazo, S. H. W. Scheres "Chapter Ten - An Introduction to Maximum-Likelihood Methods in Cryo-EM" in Methods Enzymol., G. J. Jensen, Eds. (Academic Press, 2010), pp. 263-294.
